# Supplementary material for: Engineering the Yeast Saccharomyces cerevisiae for the Production of L-(+)-Ergothioneine
Source: Front Bioeng Biotechnol. 2019 Oct 11;7:262. doi: 10.3389/fbioe.2019.00262 (PMC6797849; doi:10.3389/fbioe.2019.00262)
Supplement: Supplementary file 1 [file Table_1.DOCX]

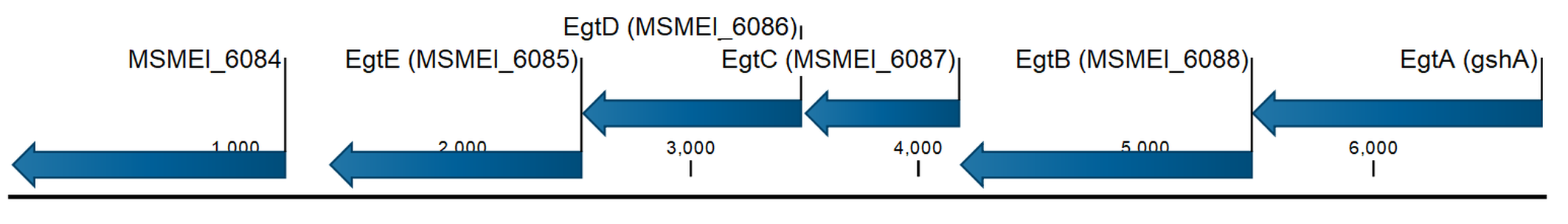


**Supplementary figure 1:** Gene cluster of ergothioneine producing genes in *Mycobacterium smegmatis*, together with MsMEI_8064, the putative ergothioneine transporter.


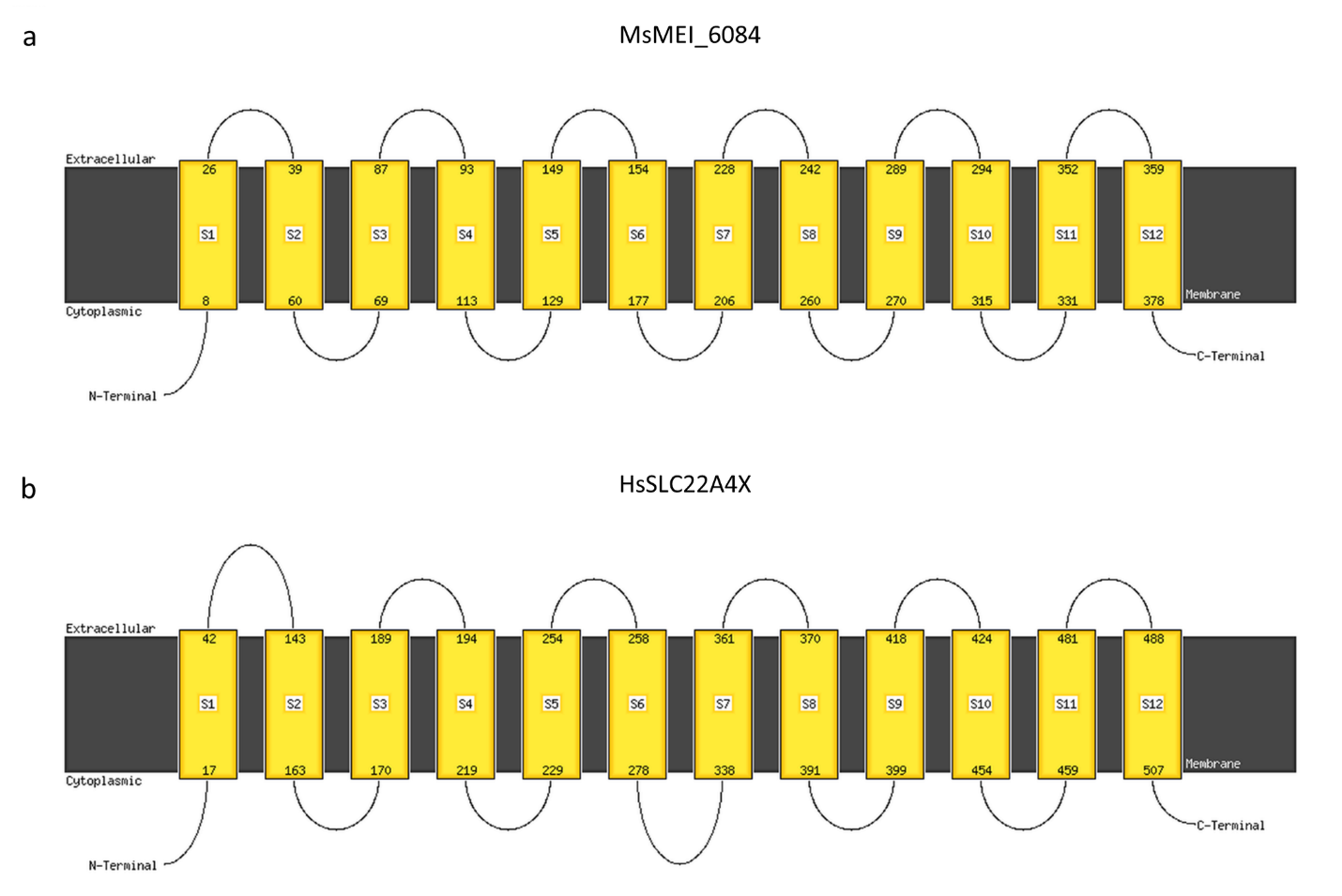


**Supplementary figure 2:** Transmembrane domain prediction by Phyre2 for **(A)** MsMEI_6084 and **(B)** HsSLC22A4X.


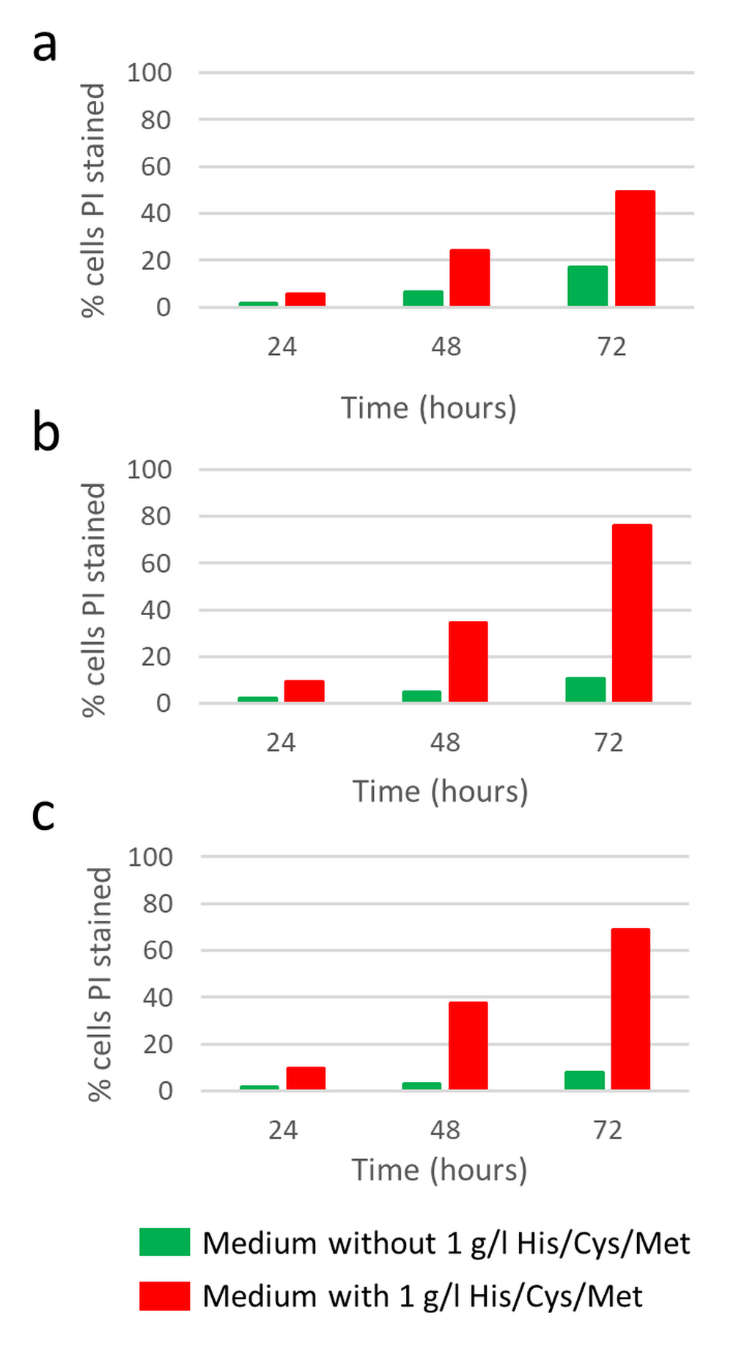


**Supplementary figure 3:** Percentage of PI stained cells for control, production strain and production strain with the transporter in media without 1 g/l histidine, cysteine and methionine versus media with 1 g/l histidine, cysteine and methionine, **(A)** Strain ST7574, **(B)** Strain ST8461, **(C)** Strain ST8654.

**Supplementary table 1:** DNA sequences and sources of the genes used in the study.

| **Protein / GenBank ID** | **DNA sequence** | **DNA source** |
| --- | --- | --- |
| MsEgtA/ AFP42520.1 | ATAATAAAAACAATGGCTTTGCCAGCTAGATCTGATTCTGGCTGTGCCGTTCCAGTCGAGTTCACTTCTGCTGAACAAGCTGCTGCCCATATTGGTGCTAACTCTTTACAAGATGGTCCAATTGGTCGTGTTGGTCTGGAAATTGAAGCTCACTGTTTCGATCTGTCTAATCCAACTCGTAGACCATCTTGGGATGAATTGTCTGCTGTCATTGCTGATGTTCCTCCATTGCCAGGAGGTTCTAGAATAACAGTGGAACCCGGAGGTGCAGTTGAATTGTCTGGTCCACCATATGATGGTCCATTGGCTGCTGTTGCTGCTTTACAAGCTGACAGGGCCGTCTTGAGGGCTGAATTTGCTAGAAGAAATTTAGGCTTGGTCTTGTTAGGTACAGATCCATTGAGACCAACGAGAAGAGTGAACCCAGGTGCTAGATATTCTGCTATGGAGCAGTTCTTCACTGCATCAGGTACTGCTGAGGCTGGTGCCGCTATGATGACTGCTACTGCATCTGTCCAAGTTAATTTGGATGCTGGTCCAAGAGATGGTTGGGCCGAGAGAGTTAGATTGGCTCATGCTTTAGGTCCCACCATGATCGCCATTACTGCTAATTCTCCAATGCTAGGTGGTCAATTTACCGGTTGGTGTTCTACAAGACAAAGAGTTTGGGGGCAATTGGATTCTGCTAGATGTGGTCCCGTTTTAGGTGTTGATGGCGACGATCCAGCCTCAGAATGGGCCAGATATGCTTTGAGAGCTCCAGTGATGTTAGTGAATTCTCCAGATGCTGTACCAGTTACTAACTGGGTCCCATTCGCTGATTGGGCTGATGGGAGAGCTGTCTTGGGTGGTAGAAGACCAACTGAAGCTGACTTGGATTATCATTTAACTACTTTATTTCCTCCAGTTAGGCCACGGAGATGGTTAGAAATTAGATATTTAGACTCGGTTCCCGACGCTTTATGGCCAGCTGCAGTTTTCACTTTAACTACTTTGTTGGATGATCCAGTTGCAGCAGAATCTGCTGCGGAAGCTACTAGACCAGTAGCTACTGCTTGGGATCGTGCTGCTAGAATGGGTTTAACTGATAGACATTTACACACCGCGGCTTTAACTTGTGTAAGATTAGCTGCTGAAAGAGCTCCGGCTGAATTGGAAGAATCTATGACATTATTAATGAGATCTGTTCAACAAAGACGGTCACCAGCTGATGATTTTTCCGATAGAGTTGTTGCTAGGGGTATCGCTGCCGCAGTTAGAGAATTGGCAAAAGGTGAATTGTGAATCTCTACTCTCTCT | Synthetic gene codon-optimized for *Saccharomyces cerevisiae* |
| MsEgtB/ WP_011731158.1 | ATGATTGCCAGAGAAACTTTGGCTGATGAATTGGCTTTGGCTAGAGAAAGAACTTTGAGATTGGTTGAATTCGATGATGCCGAATTGCATAGACAGTACAATCCATTGATGTCTCCATTGGTTTGGGACTTAGCTCATATTGGTCAACAAGAGGAATTGTGGTTGTTGAGAGATGGTAATCCAGATAGACCAGGTATGTTGGCTCCTGAAGTTGATAGATTATACGATGCCTTCGAACATTCCAGAGCTTCTAGAGTTAATTTGCCATTATTGCCACCATCTGATGCTAGAGCTTATTGTGCTACTGTTAGAGCTAAAGCTTTGGATACCTTGGATACTTTGCCAGAAGATGATCCAGGTTTTAGATTCGCCTTGGTTATCTCTCACGAAAATCAACATGACGAAACCATGTTGCAAGCCTTGAATTTGAGAGAAGGTCCACCATTATTGGATACTGGTATTCCATTGCCAGCTGGTAGACCTGGTGTTGCTGGTACTTCTGTTTTGGTTCCAGGTGGTCCATTTGTTTTGGGTGTTGATGCTTTGACTGAACCACATTCTTTGGATAACGAAAGACCAGCTCATGTTGTTGATATCCCATCTTTCAGAATTGGTAGAGTTCCAGTTACTAATGCTGAATGGCGTGAATTCATTGATGATGGTGGTTATGATCAACCTAGATGGTGGTCACCTAGAGGTTGGGCTCATAGACAAGAAGCTGGTTTGGTTGCTCCACAATTTTGGAATCCAGATGGTACTAGAACTAGATTCGGTCACATTGAAGAAATCCCAGGTGATGAACCAGTTCAACATGTTACTTTTTTCGAAGCTGAAGCTTATGCTGCTTGGGCTGGTGCTAGATTGCCAACTGAAATTGAATGGGAAAAAGCTTGTGCTTGGGATCCAGTTGCTGGTGCTCGTAGAAGATTTCCATGGGGTTCTGCTCAACCATCTGCTGCTTTAGCTAACTTAGGTGGTGATGCAAGAAGGCCAGCTCCAGTTGGTGCTTATCCAGCTGGTGCATCTGCTTATGGTGCTGAACAAATGTTGGGTGATGTTTGGGAATGGACATCTTCTCCATTAAGACCATGGCCTGGTTTTACTCCAATGATCTACGAAAGATACTCTACCCCATTCTTCGAAGGTACTACTTCTGGTGATTACAGAGTTTTGAGAGGTGGTTCATGGGCTGTTGCTCCAGGTATTTTAAGACCTTCTTTTAGAAACTGGGATCACCCAATTAGAAGGCAAATCTTTTCAGGTGTTAGATTGGCTTGGGATGTCTGA | Synthetic gene codon-optimized for *S. cerevisiae* |
| MsEgtC/ WP_011731157.1 | ATGTGCAGACATGTTGCTTGGTTGGGTGCTCCAAGATCTTTGGCTGATTTGGTTTTGGATCCACCACAAGGTTTGTTGGTTCAATCTTATGCTCCTAGAAGGCAAAAACACGGTTTGATGAATGCTGATGGTTGGGGTGCTGGTTTTTTTGATGATGAAGGTGTTGCTAGAAGATGGCGTTCTGATAAGCCTTTGTGGGGTGATGCTTCTTTTGCTTCTGTTGCTCCAGCTTTGAGATCTAGATGTGTTTTGGCTGCTGTTAGATCTGCTACTATTGGTATGCCAATTGAACCATCTGCTTCAGCTCCATTTTCTGATGGTCAATGGTTGTTGTCTCATAACGGTTTGGTTGATAGAGGTGTTTTGCCATTGACTGGTGCTGCTGAATCTACTGTTGATTCTGCTATAGTTGCTGCCTTGATTTTCTCTAGAGGTTTGGATGCTTTGGGTGCTACAATTGCTGAAGTTGGTGAATTAGATCCAAACGCCAGATTGAATATTTTGGCCGCTAATGGTTCTAGGTTGTTGGCTACTACTTGGGGTGATACTTTGTCTGTTTTACACAGACCAGATGGTGTTGTTTTAGCTTCTGAACCATATGATGATGATCCAGGTTGGTCTGATATTCCAGATAGACACTTGGTTGATGTTAGAGATGCTCATGTTGTTGTTACCCCATTGTGA | Synthetic gene codon-optimized for *S. cerevisiae* |
| MsEgtD/ WP_011731156.1 | ATGACTTTGTCCTTGGCTAATTACTTGGCTGCTGATTCTGCTGCTGAAGCTTTGAGAAGAGATGTTAGAGCTGGTTTGACTGCTGCTCCAAAATCTTTGCCACCAAAATGGTTTTATGATGCCGTTGGTTCTGATTTGTTCGATCAGATTACTAGATTGCCAGAGTACTACCCAACTAGAACTGAAGCTCAAATTTTGAGAACCAGATCCGCTGAAATTATTGCTGCTGCTGGTGCTGATACTTTGGTTGAATTAGGTTCTGGTACTTCCGAAAAGACCAGAATGTTGTTGGATGCTATGAGAGATGCCGAATTGCTGAGAAGATTCATTCCATTTGATGTTGATGCCGGTGTTTTGAGATCAGCTGGTGCAGCTATTGGTGCTGAATATCCAGGTATTGAAATTGATGCTGTTTGCGGTGATTTCGAAGAACATTTGGGTAAGATTCCACACGTTGGTAGAAGATTGGTTGTTTTCTTGGGTTCTACCATTGGTAATTTGACTCCAGCTCCAAGAGCTGAATTTTTGTCTACTTTGGCTGATACCTTGCAACCAGGTGATTCTTTGTTGTTGGGTACTGATTTGGTTAAGGATACCGGTAGATTGGTTAGAGCTTATGATGATGCAGCTGGTGTTACAGCTGCTTTTAATAGAAATGTTTTGGCCGTCGTCAACAGAGAATTGTCTGCTGATTTTGATTTGGATGCCTTCGAACATGTTGCTAAGTGGAATTCTGATGAAGAAAGGATCGAAATGTGGTTGAGAGCTAGAACTGCTCAACATGTTAGAGTTGCTGCATTGGATTTGGAAGTTGATTTTGCCGCTGGTGAAGAAATGTTGACTGAAGTTTCTTGTAAGTTCAGGCCAGAAAACGTTGTTGCTGAATTGGCTGAAGCTGGTTTAAGACAAACTCATTGGTGGACTGATCCTGCTGGTGATTTTGGTTTGTCTTTGGCTGTTAGATAA | Synthetic gene codon-optimized for *S. cerevisiae* |
| MsEgtE/ ABK70212.1 | AAAACAATGATGTTGGCTCAACAATGGAGAGATGCTAGACCAAAAGTCGCCGGTTTGCACTTAGATTCTGGTGCTTGCTCTAGACAATCTTTTGCCGTTATTGACGCAACTACTGCTCATGCTAGGCATGAAGCAGAAGTTGGTGGTTATGTTGCAGCTGAAGCCGCTACTCCAGCTTTAGATGCTGGTAGGGCTGCTGTCGCCTCTTTGATTGGTTTTGCTGCATCAGATGTTGTTTACACTTCTGGTTCTAATCACGCTATTGATTTACTATTGTCTTCTTGGCCAGGTAAAAGAACTTTAGCCTGTTTGCCCGGTGAATATGGTCCAAATTTGTCTGCTATGGCTGCAAATGGTTTTCAAGTTAGAGCTCTGCCAGTGGATGATGATGGTAGAGTTTTGGTTGATGAAGCTTCTCATGAATTGTCTGCTCATCCAGTTGCCTTAGTCCATTTGACCGCTTTGGCTTCTCATAGAGGTATTGCCCAGCCAGCAGCTGAATTGGTTGAAGCTTGTCATAACGCCGGTATCCCAGTTGTTATTGATGCTGCACAGGCATTGGGCCATTTAGATTGTAATGTTGGTGCTGATGCGGTCTATTCCTCCTCTAGAAAATGGTTGGCTGGTCCAAGGGGTGTTGGTGTACTAGCTGTTAGACCAGAATTAGCTGAAAGATTACAACCAAGAATTCCACCATCTGATTGGCCAATCCCAATGTCTGTTTTGGAAAAATTGGAATTAGGTGAGCATAACGCTGCTGCTAGAGTTGGTTTTTCTGTTGCTGTGGGTGAACATCTCGCAGCTGGACCAACTGCTGTCAGGGAAAGATTAGCTGAAGTTGGTAGATTATCTAGGCAAGTCTTGGCTGAAGTTGATGGATGGAGAGTCGTCGAACCAGTTGATCAACCAACTGCAATTACTACTTTAGAATCTACCGATGGTGCAGATCCAGCTTCTGTTAGATCTTGGTTAATCGCTGAAAGAGGTATTGTTACTACTGCTTGTGAGTTGGCTAGAGCTCCATTTGAAATGAGAACTCCAGTCCTGAGAATTTCTCCACATGTTGACGTTACAGTTGATGAATTAGAACAATTTGCTGCAGCTTTGAGAGAAGCTCCATGAAAAA | Synthetic gene codon-optimized for *S. cerevisiae* |
| NcEgt1/ XP_956324.3 | ATGCCATCTGCTGAATCTATGACTCCATCTTCTGCTTTGGGTCAATTGAAAGCTACTGGTCAACATGTCTTGTCCAAGTTGCAACAACAAACTTCCAACGCCGATATCATCGATATTAGAAGAGTTGCCGTTGAGATCAACTTGAAAACCGAAATTACCTCCATGTTCAGACCAAAAGATGGTCCAAGACAATTGCCAACCTTGTTGTTGTATAACGAAAGAGGCTTGCAGTTGTTCGAAAGAATTACTTACTTGGAAGAGTACTACTTGACCAACGACGAGATTAAGATTTTGACTAAGCACGCTACTGAAATGGCCTCTTTTATTCCATCTGGTGCCATGATTATCGAACTAGGTTCTGGTAATTTGAGGAAGGTCAACTTGTTGTTAGAAGCTTTGGATAATGCTGGTAAGGCCATTGATTATTACGCCTTGGATTTGTCCAGAGAAGAATTGGAAAGAACCTTGGCTCAAGTCCCATCTTACAAACATGTTAAGTGTCATGGTTTGTTGGGTACTTACGATGATGGTAGAGATTGGTTGAAAGCTCCAGAAAACATCAACAAGCAAAAGTGCATACTGCATCTGGGTTCTTCTATTGGTAACTTCAATAGATCTGATGCTGCCACTTTTTTGAAGGGTTTCACTGATGTTTTGGGTCCAAACGATAAGATGTTGATTGGTGTTGATGCTTGTAACGATCCAGCTAGAGTTTACCATGCTTACAATGATAAGGTTGGTATCACCCACGAATTCATCTTGAATGGTTTGAGAAACGCCAACGAAATTATTGGTGAAACCGCTTTCATTGAAGGTGATTGGAGAGTTATCGGTGAATACGTTTATGATGAAGAAGGTGGTAGACATCAAGCTTTTTATGCTCCAACTAGAGATACCATGGTTATGGGTGAATTGATCAGATCCCATGACAGAATCCAAATCGAACAGTCTCTGAAGTACTCCAAAGAGGAATCTGAAAGATTGTGGTCTACTGCTGGTTTGGAACAAGTTTCTGAATGGACTTACGGTAATGAATACGGTTTACATTTGTTGGCCAAGTCCAGAATGTCCTTCTCATTGATTCCATCAGTTTACGCTAGATCTGCTTTGCCAACTTTGGATGATTGGGAAGCTTTGTGGGCTACTTGGGATGTTGTTACTAGACAAATGTTGCCACAAGAGGAATTATTGGAGAAGCCAATCAAGTTGAGAAATGCCTGCATTTTCTACTTGGGTCATATCCCAACTTTCTTGGATATTCAGTTGACTAAGACTACCAAGCAAGCTCCATCTGAACCAGCTCATTTCTGTAAGATTTTCGAAAGGGGTATCGATCCAGATGTTGACAATCCAGAATTGTGTCATGCCCATTCTGAAATTCCAGATGAATGGCCACCAGTTGAAGAAATTTTGACTTACCAAGAAACCGTCAGATCTAGATTGAGAGGTCTATATGCTCATGGTATTGCCAACATTCCAAGAAATGTCGGTAGAGCTATTTGGGTTGGTTTCGAACATGAATTGATGCACATCGAGACTCTGTTGTACATGATGTTGCAATCTGACAAGACCTTGATTCCAACTCATATTCCAAGACCAGATTTCGATAAGTTGGCTAGAAAAGCCGAATCAGAAAGGGTTCCAAATCAATGGTTTAAGATCCCAGCTCAAGAAATCACTATTGGTTTGGATGACCCTGAAGATGGTTCCGATATTAACAAACATTACGGTTGGGATAACGAGAAGCCACCAAGAAGAGTTCAAGTTGCTGCTTTTCAAGCTCAAGGTAGACCAATTACAAACGAAGAATACGCCCAATACTTGTTGGAAAAGAACATTGATAAGTTGCCAGCTTCTTGGGCTAGATTGGATAACGAAAACATTTCTAACGGCACCACCAATTCTGTTTCTGGTCATCATTCTAACAGAACCTCCAAACAACAACTGCCATCTTCATTCTTGGAAAAAACTGCTGTTAGAACCGTTTACGGTTTGGTTCCATTGAAACATGCTTTGGATTGGCCAGTTTTTGCTTCCTATGATGAATTGGCTGGTTGTGCTGCTTATATGGGTGGTAGAATTCCAACTTTCGAAGAAACCAGATCTATCTACGCTTATGCTGATGCTCTGAAGAAGAAGAAAGAAGCTGAAAGACAACTGGGTAGAACTGTTCCAGCTGTTAATGCTCATTTGACTAACAACGGTGTTGAAATTACTCCTCCATCATCACCATCATCTGAAACTCCAGCAGAATCTTCTTCACCATCTGATTCTAACACTACCTTGATTACCACCGAGGATTTGTTCTCTGATTTGGATGGTGCTAATGTTGGTTTCCATAATTGGCATCCAATGCCTATTACTTCTAAGGGTAATACCTTGGTCGGTCAAGGTGAATTAGGTGGTGTTTGGGAATGGACATCTTCCGTTTTGAGAAAATGGGAAGGTTTTGAGCCAATGGAATTATACCCAGGTTACACTGCTGATTTCTTTGACGAAAAGCACAACATCGTTTTAGGTGGTTCATGGGCTACTCATCCAAGAATTGCTGGTAGAAAGTCTTTTGTCAACTGGTATCAAAGAAACTACCCATATGCATGGGTTGGTGCTAGAGTTGTTAGAGATTTGTGA | Synthetic gene codon-optimized for *S. cerevisiae* |
| NcEgt2/ XP_001728131.1 | ATGGTTGCTACTACTGTTGAATTGCCATTGCAACAAAAAGCTGATGCTGCTCAAACTGTTACTGGTCCATTGCCATTTGGTAACAGCTTGTTGAAAGAATTCGTTTTGGATCCAGCCTACAGAAACTTGAATCATGGTTCTTTTGGTACTATCCCATCCGCTATTCAACAGAAGTTGAGATCTTATCAAACTGCTGCTGAAGCTAGACCATGTCCATTTTTGAGATATCAAACCCCAGTTTTGTTGGACGAATCTAGAGCTGCTGTTGCTAATTTGTTGAAGGTTCCAGTTGAAACCGTTGTTTTCGTTGCTAATGCTACTATGGGTGTCAACACTGTTTTGAGAAATATCGTTTGGTCTGCTGATGGTAAGGACGAAATCTTGTACTTTGATACAATCTACGGTGCTTGCGGTAAGACCATTGATTATGTTATCGAAGATAAGAGGGGCATCGTTTCCTCTAGATGTATTCCATTGATATACCCAGCCGAAGATGATGATGTTGTTGCAGCTTTTAGAGATGCCATCAAGAAGTCTAGAGAAGAAGGTAAAAGACCAAGATTGGCCGTTATCGATGTTGTTTCTTCTATGCCAGGTGTTAGATTCCCATTCGAAGATATCGTTAAGATCTGCAAAGAGGAAGAGATCATTTCTTGCGTTGATGGTGCTCAAGGTATTGGTATGGTTGATTTGAAGATTACCGAAACCGATCCAGACTTCCTGATTTCTAATTGTCATAAGTGGTTGTTCACCCCAAGAGGTTGTGCTGTTTTTTATGTTCCAGTCAGAAACCAGCACTTGATCAGATCTACTTTGCCAACTTCTCATGGTTTCGTTCCACAAGTTGGTAATAGATTCAATCCATTGGTTCCAGCTGGTAACAAGTCTGCTTTTGTTTCTAACTTCGAATTCGTTGGTACTGTCGATAACTCTCCATTCTTCTGTGTTAAGGATGCTATTAAGTGGCGTGAAGAGGTTTTAGGTGGTGAAGAAAGAATTATGGAGTACATGACTAAGTTGGCTAGAGAAGGTGGTCAAAAGGTTGCTGAAATTTTGGGTACTAGAGTCTTGGAAAACTCTACCGGTACATTGATTAGATGCGCCATGGTTAATATTGCCTTGCCTTTTGTTGTTGGTGAAGATCCAAAAGCTCCAGTTAAGTTGACCGAAAAAGAAGAAAAAGACGTCGAAGGCTTGTACGAAATTCCACATGAAGAGGCTAATATGGCTTTCAAGTGGATGTACAACGTATTGCAAGATGAGTTCAATACCTTCGTTCCAATGACCTTTCATAGACGTAGATTTTGGGCTAGATTGTCCGCTCAAGTTTACTTGGAAATGTCTGATTTTGAATGGGCTGGCAAGACCTTAAAAGAATTGTGTGAAAGGGTTGCTAAGGGCGAGTACAAAGAATCTGCTTAA | Synthetic gene codon-optimized for *S. cerevisiae* |
| CpEgt1/ CCE33591.1 | AAAAAAAACAATGACTGCCGTTAAGCAAATTCCTGAAAGAAAGGTGTTGATAGATTCAAATCATAAGTCTCCATCAAAACCGGGTAAACATCCTAATTCTGTCATTGATATCAGGTCTAATAAGGACGATTTAAATTTACGTCATGCCCTAGTCTCATCTTTTAATCCACACGATGGAAAACCTAGGTGGCTACCTACTATGTTATTGTACGACGAAAAAGGTTTACAATTGTTTGAAGATATAACTTACTTAGATGAGTATTATTTGACTGGCTACGAAATTGAATTATTGAAGAAACATTCAGCAGAAATTGCAGCTGCTATTCCTGATGGTTCTATGGTCATCGAATTGGGCTCTGGTAATTTGAGAAAGATCTGTTTGTTGTTACAAGCCTTTGAGGATTCACATAAGTCTATCGACTACTATGCATTAGATTTATCACAAAAGGAATTAGAAAGAACTTTGAGCCATGTTCCTGACTTTAAATATGTCTCTTGTCATGGACTGCTAGGTACATATGATGATGGTGTTACATGGTTGAAACAACCAGGTATAGTCAATAAGACTAAGTGCATCATCCATCTTGGTTCGTCTATTGGGAATTTTCATAGAAATGAAGCTGCCGATTTCCTGCAGACATTTGCTGATGTAATGAAACCAGACGACTCTATGGTTATTGGTCTTGATTCATGCGGTAATCCAGAGATGTCTCGCATTCAAAGATTCATTTTGAACGGCTTATCCAATGCTAATAGCGTTTATGGCAAGGAAATATTCTATGTTCCAGATTGGAGAGTAATTGGTGAATATGTTTACGATGATGAAGGTGGCAGACACCAGGCTTTTATTTCACCTTTGAAAGAAGTCACTGCTTTAGGGTCTGTTATTAAAGCCCATGAAAGAATTAAAATTGAACAATCTTTGAAGTACTCTAAGGCCTCAGCTGACGATTTATGGAGAAATGCTGGCTTTCGAGAAACTCAAACTTGGACGAGAAACGGTGAATATGGACTACATATGTTGCAAAGAGCTGATCCGCCCTTCTCTAAGGCTCCTTCTTTGTATGCAGCTAATACTCTTCCCTCTCTTTCTGATTGGAGAGCATTGTGGTGTGCCTGGGATATTGTCACTAGAGCTATGTTGCCACAACAGGAATTGACTGAGAAACCTATAGAGTTAAGACATGCCTACATCTTTTACCTTGGTCATATTCCTACCTTCTTAGACATCCAGTTAACCAAAACATCAGCATGGGCTCCAACCTCTCCAGTTTCTTATCATGCCATTTTCGAGCGCGGCATTGATCCCGATGTTGATAACCCAGAAAAGTGTCATGATCACTCAGAGATTCCAGATGAATGGCCACCAGTCGAAGAAATTATTGCTTATCAAGATAGGGTGCGTGTTAGATTGACAGAACTGTATAAACAGGGTGTGCACACAATTACAAGAAAGGCTGCTAGAGCTATCTGGGTTTCATTTGAACATGAAGCTATGCATTTGGAAACCTTGTTGTATATGATGCTACAAAGTGATAAAGTGTTGCCACCTCCACACACTGGCGTTCCAGACTTTGAAAGAATGGCAACTAAGGCTTTCGAAGCTCGTACGCAAAATATGTGGTTCGAAATTCCAGAACAGACTATTAGTCTTGGAACAGATGATCCAGAAGATGGGGATGAAGACGTTCATTTTGGATGGGACAACGAAAAACCAGTTAGAAGAGTTAAGGTTCACGCGTTGCAAGCTCAAGGAAGACCAATTACAAATGAGGAATACGCATTATATATTTACCATACCAACTCTTCTAAACTGCCAGCATCTTGGAGTTCGTCCCCTTCATCTTCTCTGTCTAACGGCGTGTCTCATCCCAGCTCCCATAACAAGCATATTCCAACTGATTTGCCTCATTCCTTCTTGCAAGGTAAGTTTGTTAGAACCGTATATGGTTTGATACCTTTATCTTTGGCGTTGGATTGGCCTGTTCAAGCTTCTTATGATGAATTAGCTGACTGTGCATTATGGATGGGTGGAAGAATTCCAACCTTAGAGGAAGCCAGATCAATCTATGCCTTTGTTGAATCTAAAACGCAAATAGCAACAGGTAACACATTGGTCAAGAAAGTTCCTGCTGTTAATGGACACTTGGTTAATAACGGAGTTGAGGAAACTCCACCACATGAATCCTCTTCGGCAGTTGAGAATTCTTTATTCATCGACTTAGCCGGTTTGAACGTGGGTTTTAAAAGTTGGAATCCTGAACCTGTTACATCTTCTGGTACGTCTTTGGCTGGACAATCCTCTATGGGTGGTGTATGGGAGTGGACCTCTTCTGTTTTAAGACCACATGAAGGGTTCCACCCAATGGAGTTGTATCCTGGTTATACAGCCGATTTCTTTGATGAAAAACATAATATTGTTCTCGGAGGATCATGGGCTACTCATCCAAGAATAGCGGGTAGAAAAAGCTTTGTTAACTGGTATCAAAGAAACTATCCGTACGCCTGGGCTGGTGCCAGACTTGTTAAAGATGCTTGAAAAA | Synthetic gene codon-optimized for *S. cerevisiae* |
| CpEgt2/ CCE33140.1 | ATGGGTTTGTTGGAAGGTGAAGAATTGGTTTTGAGAGGTAGAGGTCAAGGTGGTGAACCTAGACCAGAAAGAGAACCAGAATTGAAGTTGGAACACGTTCCAGAAAGGGCTCCAGATGGTGAACCAGAAACTGAAGGTCAATTGGGTCCAAGAAAAGAACCTGAACATAAGTTGGAAGCTGAATCCGAACCATTGCAAGAAACTCCACAAAGAGAAGTTTTGGCTTTTGGTAGAGCTTGGAAGTCCGAATTTTTGTTTGATCCAGCTTGGAGAAACTTGAACCATGGTAGTTTTGGTACTTACCCCTTGTACATCAGAGATAAGTTGAGAGCTTATCAAGATCAAGCTGAAGCTAGACCTGATCACTTCATTAGATACGAAGAGTCCAAGTTGTTGCATAGATCTAGAGCTGCTGTTGCTAAGATAGTTAATGCTCCATTGGATACCGTTGTTTTCGTTGGTAATGCTACTGAAGGTGTCAACACTGTCTTGAGAAATTTGAGATGGGACTCCTTGGAAAAAGGTGGTCAAAAGGATGTTATCCTGTCTTTCTCTACTGTTTACGAAGCTTGTGGTAACGCTGCTGATTATATCGTTGAATACTTTGCCGGTAAGGTTGAACATAGAACCATCGAATTGGAATACCCAGTTGAAGATGCTGATGTTATTGCTGCTTTAAGAGGTGCTGCTACTCAAGTTGCTAGAGAAGGTAAAAGGGCTAGATTGGCTATGATGGATGTTGTTACTTCTAGACCAGGTGTTGTTTTTCCATGGGAAGCTGCAGTTAGAGTATGTAGAGAATTGGGTATCTTGTCCTTGGTTGATGGTGCTCAAGGTGTTGGTATGGTTAGATTGGATTTGACTGCTGCTGATCCAGATTTCTTCGTTTCTAACTGTCATAAGTGGTTGTTGGTTCCAAGAGGTTGTGCTATGTTGTATACTCCAGCTAGAACTCAATGTTTGTTGAGAACTGCTTTGGCTACTTCTCATGGTTATGTTCCACCATCTGCTGCTCCAGCTCCACCAGGTTCTAAATCTAGATATGTTGCTAACTTCGAATTCGTTGGCACTAGAGATAATGGTCCATATTTGTGTGTTGCTGATGCAATTGCTTGGAGAGAACGTGTTTGTGGTGGTGAAGAAAACATCTTGAGATACTTGTGGGCTTTGAACAAGAAGGGTATTAGAATTGTCGCTAGAGCTTTGGGTACTACCCATTTGGATAACGAAACTGAAACTTTGACCAACTGTGCTATGGGTAATGTTGCTTTGCCAATGAGAGTTGATGATGAAGATGCCTCTACTGCTTTAGATGCTGCTCCTTCTGCTGCTATTGCTGCACCAGATGTTGTTGTTGCAAGAGAAAATGTTGCATTGGTTGACAAGTGGATGAGAGAAAGATTATTCGATGACTACAAGACCTTCATGACCTTGTTCGTTATGCAAGATAGATACTGGGTTAGACTGTCTGCTCAAATCTACTTGGATGAACAAGATTATGAAGCCGCCGGTGATATTTTGAAAGCTTTGTGTGAAAGAATCAGGCGTAGAGAATATTTGGTTCCACAACCAGTTGAGTAA | Synthetic gene codon-optimized for *S. cerevisiae* |
| SpEgt1/ NP_596639.2 | ATGACAGAAATAGAAAACATTGGCGCATTAGAAGTTCTCTTCTCTCCTGAATCCATCGAGCAGAGCCTCAAACGGTGTCAACTCCCCTCCACTTTATTATACGATGAAAAAGGTTTACGACTGTTTGATGAGATTACGAATTTAAAAGAATACTACCTGTATGAAAGTGAGCTTGATATTCTGAAGAAGTTCAGCGATTCCATTGCCAACCAGTTACTGTCTCCAGATCTTCCTAACACGGTTATAGAATTAGGGTGTGGAAATATGCGCAAAACAAAACTTCTTTTAGATGCGTTTGAAAAGAAGGGCTGTGATGTGCATTTTTACGCCCTTGACCTTAATGAAGCCGAGTTGCAAAAAGGACTGCAGGAGCTTCGTCAAACTACCAATTATCAGCATGTTAAGGTGTCTGGTATTTGCGGTTGCTTTGAAAGATTGCTACAATGTTTGGACAGGTTTCGTAGTGAGCCCAATAGTCGAATTAGCATGTTGTACTTGGGTGCTTCGATTGGTAATTTTGATAGGAAATCCGCAGCATCATTTTTACGTTCGTTTGCCAGTCGTTTGAATATTCATGACAACCTTTTAATCTCCTTCGATCATAGAAACAAGGCTGAGCTAGTCCAACTAGCTTACGATGATCCTTATCGTATTACTGAAAAGTTTGAAAAGAATATTTTGGCTAGTGTCAATGCGGTTTTTGGTGAAAACCTTTTCGACGAAAATGATTGGGAATATAAAAGTGTCTACGATGAAGATCTCGGTGTTCATAGGGCCTACTTACAAGCCAAAAATGAAGTTACTGTTATTAAGGGTCCAATGTTTTTTCAATTTAAACCTAGTCATTTAATTTTGATCGAAGAAAGTTGGAAGAATAGCGATCAAGAATGTCGTCAAATCATTGAGAAAGGTGATTTTAAATTAGTCTCTAAGTATGAAAGTACGATTGCAGATTACTCGACCTATGTTATTACCAAACAATTTCCTGCTATGCTTCAACTCCCTCTTCAGCCTTGTCCTTCGTTAGCAGAATGGGATGCTCTACGCAAAGTATGGCTTTTTATTACAAATAAATTGCTTAACAAAGATAACATGTACACCGCATGGATTCCTTTGAGACATCCTCCAATTTTTTACATCGGACATGTCCCTGTTTTTAATGATATTTATCTCACAAAGATTGTCAAAAACAAAGCAACTGCTAACAAAAAACATTTTTGGGAATGGTTTCAACGTGGTATAGATCCGGACATTGAAGATCCCTCCAAGTGCCATTGGCATTCTGAAGTTCCTGAAAGCTGGCCTTCTCCTGACCAACTTCGTGAATATGAGAAAGAGTCTTGGGAATATCATATTGTAAAGTTGTGCAAAGCAATGGATGAATTGTCTACTTCTGAAAAGAGAATTCTCTGGCTTTGTTACGAACATGTAGCCATGCATGTGGAGACAACTCTTTACATCTACGTACAGTCATTTCAAAATGCAAACCAGACTGTATCAATTTGCGGATCACTTCCTGAACCAGCTGAAAAACTTACGAAAGCTCCGTTATGGGTGAATGTACCTGAAACGGAAATTGCAGTTGGTATGCCCTTGACAACACAATACACGAGTGTTGGATCAAATTTGCAATCATCCGATCTTAGTGCCCATGAAAATACAGATGAACTTTTTTATTTTGCGTGGGATAATGAGAAACCAATGAGGAAGAAACTGGTTTCTAGCTTTTCTATTGCCAATCGTCCAATTTCTAACGGTGAATATTTAGATTTTATCAATAAAAAGTCAAAAACAGAAAGGGTGTATCCAAAGCAATGGGCGGAGATTGATGGAACGCTTTACATACGAACCATGTACGGCTTATTACCCCTTGACGACTACTTGGGTTGGCCTGTTATGACTTCATACGACGATCTAAACAATTATGCGAGCTCCCAAGGATGCAGACTACCAACTGAGGATGAACTGAACTGTTTTTACGATCGGGTTCTCGAGAGAACTGATGAGCCTTATGTTAGTACCGAAGGAAAGGCAACTGGTTTTCAACAATTGCACCCTTTAGCCCTAAGTGATAATTCAAGTAATCAAATATTCACAGGAGCATGGGAATGGACAAGTACAGTTCTGGAGAAGCACGAGGATTTTGAACCTGAAGAGCTTTATCCAGATTATACACGAGATTTCTTTGATGGAAAGCATAATGTCGTTTTGGGTGGTAGCTTTGCTACGGCTACGCGCATTTCAAATAGAAGAAGCTTCAGGAACTTTTACCAAGCTGGCTATAAATATGCATGGATTGGAGCTAGACTAGTCAAAAACTAA | Genomic DNA of *Schizosaccharomyces* |
| SpEgt2/ NP_595091.1 | ATGGCTGAAAACAACGTCTACGGCCATGAAATGAAAAAGCATTTTATGCTTGATCCCGATTACGTGAATGTAAATAACGGAAGTTGTGGAACAGAATCTCTTGCTGTTTACAATAAACATGTCCAACTTTTAAAGGAAGCTCAGAGCAAGCCAGATTTTATGTGCAATGCCTATATGCCGATGTACATGGAGGCTACTCGAAATGAAGTTGCCAAGCTGATAGGCGCGGATTCAAGTAATATAGTTTTTTGCAATTCCGCTACAGATGGGATTAGTACGGTTTTGTTGACATTTCCGTGGGAACAGAATGATGAGATATTGATGCTAAATGTTGCCTATCCTACTTGTACATATGCCGCTGATTTTGCAAAGAATCAGCATAATTTACGATTAGACGTTATCGATGTTGGGGTGGAAATTGATGAAGATCTATTCCTTAAAGAAGTAGAACAGCGTTTTTTGCAGTCCAAGCCGAGAGCATTTATCTGTGATATTTTAAGTTCTATGCCCGTTATCTTGTTTCCTTGGGAAAAAGTCGTAAAGCTTTGTAAAAAGTATAATATTGTTAGCATTATTGATGGTGCTCATGCCATAGGTCATATTCCTATGAATTTGGCTAATGTTGATCCTGATTTTTTGTTTACCAATGCTCATAAATGGTTAAACTCACCAGCTGCATGCACTGTACTCTATGTCTCAGCTAAAAATCACAATCTCATCGAAGCACTTCCTCTCTCATACGGTTATGGATTAAGAGAAAAGGAATCAATTGCCGTAGATACTCTTACCAATCGGTTTGTCAATTCTTTCAAGCAAGATTTACCTAAGTTTATAGCTGTTGGTGAGGCTATTAAGTTTCGAAAATCCATTGGAGGGGAAGAAAAGATTCAACAATATTGTCATGAAATAGCTTTAAAGGGAGCCGAAATTATTTCTAAAGAACTGGGCACTTCCTTTATCAAACCTCCATACCCAGTTGCAATGGTAAACGTCGAAGTTCCCTTACGCAACATTCCCTCCATAGAAACACAGAAAGTATTTTGGCCTAAATATAATACATTCCTTCGATTTATGGAATTTAAAGGAAAATTTTACACTAGACTTAGCGGTGCGGTGTATTTAGAAGAATCAGATTTCTATTATATTGCTAAAGTTATTAAAGACTTCTGCTCTCTTTGA | Genomic DNA of *S. pombe* |
| MsMEI_6084/  AFP42515.1 | ATGCCATTTTCCCTGTACCCACTTGCAGTTGCTGTGTTCGCTATGGGAACCTCTGAATTTATGTTGGCAGGATTGGTTCCTGATATTGCAGCTGACCTCGGAGTGTCTATTGGCTCTGCTGGACTGTTGACTTCTGCTTTTGCTGTTGGAATGGTCGTGGGAGCTCCTTCTATGGCAGCTCTGACAAGAAGATGGCGCGCTAGAGTGTCTCTGAGCGCTTTTCTCCTTACCTTCGCTCTCGTGCATGTTCTGGGAGCCGTTACCACCTCTTTTGGAGTGCTGCTGGTGACAAGACTTGTGGCAGCTGTGGCTAATGCTGGATTCCTGGCCGTTGCCCTGAGTACAGCAGCAACACTGGTGCCAGCTGGAAGGCAGGGACGTGGACTTGCAGTTCTGCTTGCCGGAACAACCCTCGCAACAATTGCTGGAGTGCCAGGAGGAGCTGTGCTTGGAACAATGTTGGGATGGAGAGCAACCTTTTGGGCAATTGCTCTGCTGTGCCTGCCAGCAGTTGTTGGCATTGCAACCGCACTTCCTGCTGGCGCTGGTAGAGCCGGTTGGCCCGTTGCCGGAGCCAGTCTGTGCGATGAACTGGCCCAGCTGGGAAGAAAAAGACTGGCTCTGGCTATGCTTCTTGCTGCACTGGTCAATGCTGGAACATTTGCTACCTTTACATTTCTTGCTCCAATTGTGACAGAAAGTGCTGGACTTGGCCGCCTGTGGGTGTCTGTGGTGCTGCTTCTGTTCGGATTCGGAAGCTTTATTGGAGTGACAGTCGCCGGAAGGCTCAGCGATACAAGACCTGGAATTGTGATCGGCGCAGGTGGACCTGCTCTGCTGGCTGGATGGGCCGCTCTTGCACTTCTGTCCTCTCAGCCAGTGGCACTGTTGCCACTTGCATTCGTGCAGGGAGCACTGAGCTTTGCCGTTGGATCCACATTGATTACAAGGGTGCTGTATGAGGCATCAGCTGCCCCAACAATGGGTGGAGCTTATGCTACTGCAGCACTGAATGTCGGAGCAGCCGGTGGACCAGTCGCAGCTGCAGCCGCTCTGGGTAATCATGCAAACGTGGTGGCACCAGTTTGGGTTAGTTCCGTGATGGTGGCATTGGCTCTTCTCATCGCTGTTCCAATGCTGAAGATTGTGGCTCCAAGACCAAATCCAGCCACATCTACACCACCACTGGGAGGAAATTGCGGATAA | Synthetic gene codon-optimized for *S. cerevisiae* |
| HsSLC22A4X/  CAA71007.1 | ATGCGTGACTATGATGAGGTCATCGCATTCCTTGGCGAATGGGGACCATTCCAGCGCTTGATCTTCTTTCTGCTTAGCGCCTCTATTATCCCTAATGGCTTTAATGGTATGTCCGTTGTCTTCCTGGCCGGTACCCCTGAACATCGCTGTAGAGTGCCAGACGCCGCAAACCTGAGCAGCGCCTGGCGCAACAACTCTGTCCCTCTGAGACTGCGTGATGGCCGCGAGGTCCCCCACAGCTGTAGCCGCTACAGACTGGCCACCATCGCCAACTTCTCCGCTCTCGGACTGGAGCCAGGTCGCGATGTTGATCTGGGACAGCTGGAACAGGAGAGCTGTCTGGATGGCTGGGAGTTCAGCCAGGACGTCTACCTGTCCACCGTCGTGACCGAATGGAATCTGGTTTGTGAGGACAACTGGAAGGTGCCACTGACCACCTCCCTGTTCTTCGTTGGCGTCCTTCTGGGCTCCTTCGTGTCCGGTCAGCTGTCAGATAGATTTGGCAGGAAGAACGTTCTCTTCGCAACCATGGCTGTACAGACTGGCTTCAGCTTCCTGCAGATTTTCTCCATCAGCTGGGAGATGTTCACTGTTTTGTTTGTCATCGTGGGCATGGGCCAGATCTCCAACTATGTCGTTGCCTTCATCCTTGGAACAGAAATTCTTGGCAAGTCAGTTCGTATTATTTTCTCTACATTAGGAGTGTGCACATTTTTTGCAGTTGGCTATATGCTGCTGCCACTGTTTGCTTACTTCATCAGAGACTGGCGTATGCTGCTGCTGGCCCTGACGGTTCCTGGAGTGCTGTGTGTCCCACTGTGGTGGTTCATTCCTGAATCTCCCAGATGGCTGATCTCCCAGAGAAGATTTAGAGAGGCTGAAGATATCATCCAAAAAGCTGCAAAAATGAACAACATCGCTGTCCCAGCAGTCATTTTTGATTCTGTTGAAGAGCTGAATCCTCTGAAGCAGCAGAAAGCTTTCATTCTGGATCTGTTCAGAACTAGAAATATTGCCATTATGACCATTATGTCTTTGCTGCTTTGGATGCTGACCTCAGTGGGTTACTTTGCTCTGTCTCTGGATGCTCCTAATTTGCATGGAGATGCCTACCTGAACTGTTTCCTGTCTGCCTTGATTGAAATTCCAGCTTACATTACAGCCTGGCTGCTGTTGAGAACCCTGCCAAGGCGTTATATCATCGCTGCAGTTCTGTTCTGGGGAGGAGGTGTTCTTCTTTTCATTCAACTGGTGCCTGTCGATTATTACTTCTTGTCCATTGGTCTGGTCATGCTGGGAAAATTTGGTATCACCTCTGCTTTCTCCATGCTGTATGTCTTCACTGCTGAACTGTACCCAACCCTGGTCAGGAACATGGCTGTGGGTGTCACATCCACGGCCTCCAGAGTTGGCAGCATCATTGCCCCCTACTTTGTTTACCTCGGTGCTTACAACAGAATGCTGCCTTACATCGTCATGGGTTCTCTGACTGTCCTGATTGGAATCCTTACCCTTTTTTTCCCTGAATCCTTGGGAATGACTCTTCCAGAAACCTTAGAACAGATGCAGAAAGTGAAATGGTTCAGATCTGGAAAAAAAACAAGAGACTCAATGGAGACAGAGGAAAATCCCAAGGTTCTTATTACTGCATTCTAA | Synthetic gene codon-optimized for *S. cerevisiae* |

**Supplementary table 2:** List of primers used for cloning

| **ID** | **Name** | **Sequence 5´to 3´** |
| --- | --- | --- |
| PR-5 | pTEF1_fw | ACCTGCACUTTGTAATTAAAACTTAG |
| PR-6 | pTEF1-rv | CACGCGAUGCACACACCATAGCTTC |
| PR-1566 | <-pPGK1-pTEF1->_fw | ACCTGCACUTTGTTTTATATTTGTTG |
| PR-1567 | <-pPGK1-pTEF1->_rv | ATGACAGAUTTGTAATTAAAACTTAG |
| PR-21823 | MsEgtB_fwd | ATCTGTCAUAAAACAATGATTGCCAGAGAAACTTTGGCT |
| PR-21824 | MsEgtB_rev | CACGCGAUTCAGACATCCCAAGCCAATCTAA |
| PR-21825 | MsEgtC_fwd | ATCTGTCAUAAAACAATGTGCAGACATGTTGCTTGGTT |
| PR-21826 | MsEgtC_rev | CACGCGAUTCACAATGGGGTAACAACAACAT |
| PR-21827 | MsEgtD_fwd | AGTGCAGGUAAAACAATGACTTTGTCCTTGGCTAATTA |
| PR-21828 | MsEgtD_rev | CGTGCGAUTTATCTAACAGCCAAAGACAAACCAAAA |
| PR-21829 | CpEgt2_fwd | AGTGCAGGUAAAACAATGGGTTTGTTGGAAGGTGAAG |
| PR-21830 | CpEgt2_rev | CGTGCGAUTTACTCAACTGGTTGTGGAACCAAATATTCTCT |
| PR-21831 | NcEgt1_fwd | AGTGCAGGUAAAACAATGCCATCTGCTGAATCTATGAC |
| PR-21832 | NcEgt1_rev | CGTGCGAUTCACAAATCTCTAACAACTCTAGCACCAA |
| PR-21833 | NcEgt2_fwd | AGTGCAGGUAAAACAATGGTTGCTACTACTGTTGAATTGC |
| PR-21834 | NcEgt2_rev | CGTGCGAUTTAAGCAGATTCTTTGTACTCGCCCTTA |
| PR-21835 | SpEgt1_fwd | AGTGCAGGUAAAACAATGACAGAAATAGAAAACATTGGCGC |
| PR-21836 | SpEgt1_rev | CGTGCGAUTTAGTTTTTGACTAGTCTAGCTCCAATCCATGC |
| PR-21837 | SpEgt2_fwd | AGTGCAGGUAAAACAATGGCTGAAAACAACGTCTACG |
| PR-21838 | SpEgt2_rev | CGTGCGAUTCAAAGAGAGCAGAAGTCTTTAATAACTTTAGCAATATAATAGAAATCTGA |
| PR-22107 | MsEgtA_fwd | AGTGCAGGUAAAACAATGGCTTTGCCAGCTAGATC |
| PR-22108 | MsEgtA_rev | CGTGCGAUTCACAATTCACCTTTTGCCAATTCTCTAACTG |
| PR-22109 | MsEgtE_fwd | AGTGCAGGUAAAACAATGATGTTGGCTCAACAATGGAG |
| PR-22110 | MsEgtE_rev | CGTGCGAUTCATGGAGCTTCTCTCAAAGCTG |
| PR-22111 | CpEgt1_fwd | AGTGCAGGUAAAACAATGACTGCCGTTAAGCAAATTCC |
| PR-22112 | CpEgt1_rev | CGTGCGAUTCAAGCATCTTTAACAAGTCTGGCA |
| PR-22825 | HsSLC22A4X_MFG1_fwd | AGTGCAGGUAAAACAATGCGTGACTATGATGAGGTCATCGCATTCCTT |
| PR-22826 | HsSLC22A4X_MFG1_rev | CGTGCGAUTTAGAATGCAGTAATAAGAACCTTGGGATTTTCCTCTGTC |
| PR-22828 | MsMEI_6084_MFG1_fwd | AGTGCAGGUAAAACAATGCCATTTTCCCTGTACCCACTTGCAG |
| PR-22829 | MsMEI_6084_MFG1-rev | CGTGCGAUTTATCCGCAATTTCCTCCCAGTGGTGGT |
| PR-23559 | Tor1_gRNA | ATCCTCAGTTAACTAGCCAGGTTTTAGAGCTAGAA |
| PR-23560 | TOR1_repair_dsDNA | GTAAAGTGAAACATACATCAACCGGCTAGCAGGTTTGCATTGATTAACTGCGGTGTCATTTTTCATTTCGTGCTTTGTTTACTATTTATT |
| PR-23563 | Yih1_gRNA | AAACAGAGTCCATCACTTCCGTTTTAGAGCTAGAA |
| PR-23564 | Yih1_repair_dsDNA | TATGTAACAAGAAAAAAAAAAAAGAGAGAGGAAAGAAAAGCTCATAATCATTATGATTATGTCAAGCACCTGTAACCTTGATCACATGCG |
| PR-23861 | GFP-Nterm_fwd | AGTGCAGGUAAAACAATGTCTAAAGGTGAAGAATTATTCACTGGTGTTGTCCCA |
| PR-23862 | GFP-Nterm_rev | ATGACAGAUTTTGTACAATTCATCCATACCATGGGTAATACCAGCAG |
| PR-23863 | MsMEI_6084-Nterm_fwd | ATCTGTCAUATGCCATTTTCCCTGTACCCACTTGCA |
| PR-23864 | MsMEI_6084-Cterm_rev | ATGACAGAUTCCGCAATTTCCTCCCAGTGGTGG |
| PR-23865 | GFP-Cterm_fwd | ATCTGTCAUATGTCTAAAGGTGAAGAATTATTCACTGGTGTTGTCCCA |
| PR-23866 | GFP-Cterm_rev | CGTGCGATUTATTTGTACAATTCATCCATACCATGGGTAATACCAGCAGC |
| PR-23873 | HsSLC22A4X-Cterm_rev | ATGACAGAUTTAGAATGCAGTAATAAGAACCTTGGGATTTTCCTCTGTCTCC |
| PR-23874 | HsSLC22A4X-Nterm_fwd | ATCTGTCAUATGCGTGACTATGATGAGGTCATCGCATTCC |

**Supplementary table 3:** List of primers used for sequencing

| **ID** | **Name** | **Sequence 5´to 3´** |
| --- | --- | --- |
| PR-224 | ADH1_test_fw | GAAATTCFCTTATTTAGAAGTGTC |
| PR-225 | CYC1_test_rv | CTCCTTCCTTTTCGGTTAGAG |
| PR-21827 | MsEgtD_fwd | AGTGCAGGUAAAACAATGCCATCTGCTGAATCTATGAC |
| PR-21831 | NcEgt1_fwd | AGTGCAGGUAAAACAATGCCATCTGCTGAATCTATGAC |
| PR-21833 | NcEgt2_fwd | AGTGCAGGUAAAACAATGGTTGCTACTACTGTTGAATTGC |
| PR-22111 | CpEgt1_fwd | AGTGCAGGUAAAACAATGACTGCCGTTAAGCAAATTCC |
| PR-22277 | Seq_NcEgt1_1_fwd | TCTTTCTTCTTCTTCAGAGCA |
| PR-22278 | Seq_NcEgt1_2_fwd | TATCGAAATCTGGTCTTGGAA |
| PR-22279 | Seq_NcEgt1_3_fwd | AAAACATCAACAAGCAAAAGTGC |
| PR-22280 | Seq_NcEgt2_1_fwd | CTTAGTCATGTACTCCATAATTCTTTCTT |
| PR-22281 | Seq_SpEgt1_1_fwd | CTGTTTTTGACTTTTTATTGATAAAATCTAAATATT |
| PR-22282 | Seq_SpEgt1_2_fwd | GTTGCTTTGTTTTTGACAATCTTTGT |
| PR-22283 | Seq_SpEgt1_3_fwd | GTTTCTATGATCGAAGGAGATTAAAA |
| PR-22284 | Seq_SpEgt2_1_fwd | CCATTTATGAGCATTGGTAAACAA |
| PR-22285 | Seq_CpEgt1_1_fwd | TCTAAGGTTGGAATTCTTCCA |
| PR-22286 | Seq_CpEgt1_2_fwd | CTTCATGTTCAAATGAAACCCA |
| PR-22287 | Seq_CpEgt1_3_fwd | CTTAGAGTACTTCAAAGATTGTTCAA |
| PR-22288 | Seq_CpEgt2_1_fwd | AAACACGTTCTCTCCAAGCA |
| PR-22289 | Seq_CpEgt2_2_fwd | AACTGGGTATTCCAATTCGATG |
| PR-22290 | Seq_MsEgtB_1_rev | ACAATTCCTCTTGTTGACCAAT |
| PR-22291 | Seq_MsEgtB_2_rev | ATTGTGGAGCAACCAAACCA |
| PR-22292 | Seq_MsEgtE_1_fwd | GCTAGTACACCAACACCCCT |
| PR-22293 | Seq_MsEgtA_1_fwd | TGGAGCTCTCAAAGCATATCT |
| PR-22294 | Seq_MsEgtC_1_rev | AAAAACCAGCACCCCAACCA |
| PR-22295 | Seq_PGK1_middle_rev | AATTTCGTCACACAACAAGG |
| PR-22296 | Seq_TEF1_start_revx | TTTGAAGCTATGGTGTGTGC |
| PR-22900 | Seq_MsMEI_6084_fwd | GTAGCAAATGTTCCAGCATTG |
| PR-22901 | Seq_Hs.SLC22A4X_1_fwd | TGATATAACGCCTTGGCAGG |
| PR-22902 | Seq_Hs.SLC22A4X_2_fwd | AACGACATAGTTGGAGATCTG |
| PR-23888 | Seq_MsMEI_6084_2_fwd | GTAGCAAATGTTCCAGCATTG |
| PR-23889 | Seq_GFP_fwd | ATTAACTAAGGTATCACCTTCAAAC |
| PR-23890 | Seq_Hs.SLC22A4X_3_fwd | ACTCTACAGCGATGTTCAGG |

**Supplementary table 4:** List of BioBricks generated by PCR amplification

| **ID** | **Name** | **Primers** | **Template** |
| --- | --- | --- | --- |
| BB8 | <-pTEF1 | PR-5, PR-6 | pCfB0029 |
| BB312 | <-pPGK1-pTEF1-> | PR-1566, PR-1567 | pCfB0029 |
| BB3238 | MsEgtA | PR-22107, PR-22108 | pCfB8321 (synthetic MsEgtA gene) |
| BB3239 | MsEgtB | PR-21823, PR-21824 | pCfB8322 (synthetic MsEgtB gene) |
| BB3240 | MsEgtC | PR-21825, PR-21826 | pCfB8323 (synthetic MsEgtC gene) |
| BB3241 | MsEgtD | PR-21827, PR-21828 | pCfB8324 (synthetic MsEgtD gene) |
| BB3242 | MsEgtE | PR-22109, PR-22110 | pCfB8325 (synthetic MsEgtE gene) |
| BB3243 | NcEgt1 | PR-21831, PR-28132 | pCfB8326 (synthetic NcEgt1 gene) |
| BB3244 | NcEgt2 | PR-21833, PR-21834 | pCfB8327 (synthetic NcEgt2 gene) |
| BB3245 | CpEgt1 | PR-22111, PR-22112 | pCfB8328 (synthetic CpEgt1 gene) |
| BB3246 | CpEgt2 | PR-21829, PR-21830 | pCfB8329 (synthetic CpEgt2 gene) |
| BB3247 | SpEgt1 | PR-21835, PR-21836 | Genomic DNA from *S. pombe* |
| BB3248 | SpEgt2 | PR-21837, PR-21838 | Genomic DNA from *S. pombe* |
| BB3772 | MsMEI_6084 | PR-22828, PR-22829 | pCfB8755 (synthetic MsMEI_6084 gene) |
| BB3823 | MsMEI_6084-nterm | PR-23863, PR-22829 | pCfB8374 |
| BB3824 | MsMEI_6084-cterm | PR-22828, PR-23864 | pCfB8374 |
| BB3825 | Hs.SLC22A4X-nterm | PR-23874, PR-22826 | pCfB8375 |
| BB3826 | Hs.SLC22A4X-cterm | PR-22825, PR-23873 | pCfB8375 |
| BB3827 | yeGFP-nterm | PR-23861, PR-23862 | pCfB1914 |
| BB3828 | yeGFP-cterm | PR-23865, PR-23866 | pCfB1914 |
| BB4016 | HsSLC22A4X | PR22825, PR22826 | pCfB8388 (synthetic HsSLC22A4X gene) |

**Supplementary table 5:** List of plasmids made by USER cloning

| **ID** | **Name** | **Description** | **Template** | **Biobricks** | **Source** |
| --- | --- | --- | --- | --- | --- |
| **Basic vectors** | | | | | |
| pCfB1914 | pMeLS0025 | Plasmid carrying yeGFP for amplification of BB3827 and BB3828 |  |  | Skjoedt et al., 2016 |
| pCfB2312 | TEF1p-Cas9-CYC1t_kanMX | Episomal plasmid for Cas9 expression |  |  | Stovicek et al., 2015 |
| **Basic integrative vectors** | | | | | |
| pCfB2899 | X-2-MarkerFree | Backbone plasmid for construction of gene integration plasmids at X-2 site |  |  | Jessop-Fabre et al., 2016 |
| pCfB2903 | XI-2 | Backbone plasmid for construction of gene integration plasmids at XI-2 site |  |  | Jessop-Fabre et al., 2016 |
| pCfB3034 | X-3 | Backbone plasmid for construction of gene integration plasmids at X-3 site |  |  | Jessop-Fabre et al., 2016 |
| pCfB3039 | XII-2 | Backbone plasmid for construction of gene integration plasmids at XII-2 site |  |  | Jessop-Fabre et al., 2016 |
| **gRNA vectors** | | | | | |
| pCfB3020 | X-2 gRNA | Plasmid carrying gRNA to cut at X-2 site |  |  | Jessop-Fabre et al., 2016 |
| pCfB3042 | p-gRNA X-4 | Plasmid for gRNA at X-4 site, used as backbone to make new gRNA plasmids |  |  | Jessop-Fabre et al., 2016 |
| pCfB3043 | p-gRNA XI-1 | gRNA plasmid for targeting XI-1 site |  |  | Jessop-Fabre et al., 2016 |
| pCfB3045 | p-gRNA XI-3 | gRNA plasmid for targeting XI-3 site |  |  | Jessop-Fabre et al., 2016 |
| pCfB3051 | X-3, XI-2, XII-2 gRNA | Plasmid carrying gRNAs to cut at X-2, XI-2 and XII-2 site |  |  | Jessop-Fabre et al., 2016 |
| **Integrative vectors** | | | | | |
| pCfB8331 | pX-3-MarkerFree-NcEgt1<-TEF1 | Integration of NcEgt1 at X-3 site on *S.cerevisiae* genome | pCfB3034 | BB8, BB3243 | This study |
| pCfB8332 | pXII-2-MarkerFree-NcEgt2<-TEF1 | Integration of NcEgt2 at XII-2 site on *S.cerevisiae* genome | pCfB3039 | BB8, BB3244 | This study |
| pCfB8333 | pX-3-MarkerFree-SpEgt1<-TEF1 | Integration of SpEgt1 at X-3 site on *S.cerevisiae* genome | pCfB3034 | BB8, BB3247 | This study |
| pCfB8334 | pXII-2-MarkerFree-SpEgt2<-TEF1 | Integration of SpEgt2 at XII-2 site on *S.cerevisiae* genome | pCfB3039 | BB8, BB3248 | This study |
| pCfB8335 | pX-3-MarkerFree-CpEgt1<-TEF1 | Integration of CpEgt1 at X-3 site on *S.cerevisiae* genome | pCfB3034 | BB8, BB3245 | This study |
| pCfB8336 | pXII-2-MarkerFree-CpEgt2<-TEF1 | Integration of CpEgt2 at XII-2 site on *S.cerevisiae* genome | pCfB3039 | BB8, BB3246 | This study |
| pCfB8337 | pX-3-MarkerFree-MsEgtD<-PGK1-TEF1->MsEgtB | Integration of MsEgtD and MsEgtB at X-3 site on *S. cerevisiae* genome | pCfB3034 | BB312, BB3239, BB3241 | This study |
| pCfB8338 | pXII-2-MarkerFree-MsEgtE<-TEF1 | Integration of MsEgtE at XII-2 site on *S.cerevisiae* genome | pCfB3039 | BB8,  BB3242 | This study |
| pCfB8339 | pXI-2-MarkerFree-MsEgtA<-PGK1-TEF1->MsEgtC | Integration of MsEgtA and MsEgtC at XI-2 site on *S. cerevisiae* genome | pCfB2903 | BB312, BB3238, BB3240 | This study |
| pCfB8374 | X-2-MarkerFree-MsMEI_6084<-TEF1 | Integration of MsMEI_6084 at X-2 site on *S. cerevisiae* genome | pCfB2899 | BB8, BB3772 | This study |
| pCfB8375 | X-2-MarkerFree-Hs.SLC22A4X<-TEF1 | Integration of Hs.SLC22A4X at X-2 site on S. cerevisiae genome | pCfB2899 | BB8, BB4016 | This study |
| pCfB8800 | X-2-MarkerFree-MsMEI_6084-GFPnterm<-TEF1 | Integration of GFP-MsMEI_6084 at X-2 site on S. cerevisiae genome | pCfB2899 | BB8, BB3823, BB3827 | This study |
| pCfB8801 | X-2-MarkerFree-GFPcterm-MsMEI_6084<-TEF1 | Integration of MsMEI_6084-GFP at X-2 site on S. cerevisiae genome | pCfB2899 | BB8, BB3824, BB3828 | This study |
| pCfB8802 | X-2-MarkerFree-GFPcterm-Hs.SLC22A4X<-TEF1 | Integration of Hs.SLC22A4X-GFP at X-2 site on S. cerevisiae genome | pCfB2899 | BB8, BB3826, BB3827 | This study |
| pCfB8803 | X-2-MarkerFree-Hs.SLC22A4X-GFPnterm-TEF1 | Integration of GFP-Hs.SLC22A4X at X-2 site on S. cerevisiae genome | pCfB2899 | BB8, BB3825, BB3828 | This study |
| pCfB8804 | pXI-3-MarkerFree-NcEgt1<-pTEF1 | Integration of NcEgt1 at XI-3 site on S. cerevisiae genome | pCfB2904 | BB8, BB3243 | This study |
| pCfB8805 | pXI-1-MarkerFree-CpEgt2<-pTEF1 | Integration of CpEgt2 at XI-1 site on S.cerevisiae genome | pCfB3036 | BB8, BB3246 | This study |

**Supplementary table 6:** List of plasmids made by PCR

| **ID** | **Name** | **Description** | **Primers** | **Source** |
| --- | --- | --- | --- | --- |
| pCfB8730 | gRNA_Yih1 | gRNA plasmid targeting Yih1 for gene knock out | PR-10277,  PR-23563 | This study |
| pCfB8732 | gRNA_Tor1 | gRNA plasmid targeting TOR1 for gene knock out | PR-10277,  PR-23559 | This study |

**Supplementary table 7:** List of yeast strains

| **Strain** | **Characteristics** | **Strain specifics** | **Parent strain** | **Integration** | **Source** |
| --- | --- | --- | --- | --- | --- |
| ST1 | CEN.PK113-7D  Mata MAL2-8c SUC2 URA3 HIS3 LEU2 TRP1 | Parent strain |  |  | Peter Kötter (Goethe University, Frankfurt/Main, Germany) |
| ST7574 | CEN.PK113-7D + pCfB2312 (Cas9 plasmid) | Background strain, plasmid cured out for ERG production experiments | ST1 | pCfB2312 (no integration, episomal) | This study |
| ST8459 | NcEgt1 + NcEgt2 | Fungal pathway | ST7574 | pCfB8331, pCfB8332 | This study |
| ST8460 | NcEgt1 + SpEgt2 | Fungal pathway | ST7574 | pCfB8331, pCfB8334 | This study |
| ST8461 | NcEgt1 + CpEgt2 | Fungal pathway | ST7574 | pCfB8331, pCfB8336 | This study |
| ST8462 | SpEgt1 + SpEgt2 | Fungal pathway | ST7574 | pCfB8333, pCfB8334 | This study |
| ST8463 | SpEgt1 + NcEgt2 | Fungal pathway | ST7574 | pCfB8332, pCfB8333 | This study |
| ST8464 | SpEgt1 + CpEgt2 | Fungal pathway | ST7574 | pCfB8333, pCfB8336 | This study |
| ST8465 | CpEgt1 + CpEgt2 | Fungal pathway | ST7574 | pCfB8335, pCfB8336 | This study |
| ST8466 | CpEgt1 + NcEgt2 | Fungal pathway | ST7574 | pCfB8332, pCfB8335 | This study |
| ST8467 | CpEgt1 + SpEgt2 | Fungal pathway | ST7574 | pCfB8334, pCfB8335 | This study |
| ST8468 | MsEgtD/B + MsEgtA/C + MsEgtE | Bacterial pathway | ST7574 | pCfB8337, pCfB8338,  pCfB8339 | This study |
| ST8469 | MsEgtD/B + MsEgtA/C + NcEgt2 | Mixed pathway | ST7574 | pCfB8332, pCfB8337,  pCfB8339 | This study |
| ST8470 | MsEgtD/B + MsEgtA/C + SpEgt2 | Mixed pathway | ST7574 | pCfB8334, pCfB8337,  pCfB8339 | This study |
| ST8471 | MsEgtD/B + MsEgtA/C + CpEgt2 | Mixed pathway | ST7574 | pCfB8336, pCfB8337,  pCfB8339 | This study |
| ST8472 | NcEgt1 + MsEgtE | Mixed pathway | ST7574 | pCfB8331, pCfB8338 | This study |
| ST8473 | SpEgt1 + MsEgtE | Mixed pathway | ST7574 | pCfB8333, pCfB8338 | This study |
| ST8474 | CpEgt1 + MsEgtE | Mixed pathway | ST7574 | pCfB8335, pCfB8338 | This study |
| ST8654 | NcEgt1 + CpEgt2 + MsMEI_6084 | Fungal pathway + putative bacterial transporter from *M. smegmatis* | ST8461 | pCfB8374 | This study |
| ST8655 | NcEgt1 + CpEgt2 + HsSLC22A4X | Fungal pathway + transporter from *H. sapiens* | ST8461 | pCfB8375 | This study |
| ST8882 | NcEgt1 + CpEgt2 + MsMEI_6084 + ΔYih1 | Fungal pathway + MsMEI_6084 + Yih1 KO | ST8654 | pCfB8730,  PR-23564 | This study |
| ST8883 | NcEgt1 + CpEgt2 + MsMEI_6084 + ΔTor1 | Fungal pathway + MsMEI_6084 + Tor1 KO | ST8654 | pCfB8732,  PR-23560 | This study |
| ST8921 | NcEgt1 + CpEgt2 + GFPnterm-MsMEI_6084 | Fungal pathway + MsMEI_6084 linked to GFP at N-terminus | ST8461 | pCfB8800 | This study |
| ST8922 | NcEgt1 + CpEgt2 + MsMEI_6084-GFPcterm | Fungal pathway + MsMEI_6084 linked to GFP at C-terminus | ST8461 | pCfB8801 | This study |
| ST8923 | NcEgt1 + CpEgt2 + HsSLC22A4X- GFPcterm | Fungal pathway + HsSLC22A4X linked to GFP at C-terminus | ST8461 | pCfB8802 | This study |
| ST8924 | NcEgt1 + CpEgt2 + GFPnterm-HsSLC22A4X | Fungal pathway + HsSLC22A4X linked to GFP at N-terminus | ST8461 | pCfB8803 | This study |
| ST8925 | NcEgt1 + CpEgt2 + second copy of CpEgt2 | Fungal pathway with extra CpEgt2 | ST8461 | pCfB8805 | This study |
| ST8926 | NcEgt1 + CpEgt2 + second copy of NcEgt1 | Fungal pathway with extra NcEgt1 | ST8461 | pCfB8804 | This study |
| ST8927 | NcEgt1 + CpEgt2 + second copy of both NcEgt1 and CpEgt2 | Two copies of fungal pathway | ST8461 | pCfB8804, pCfB8805 | This study |

**Supplementary table 8:** Fractional factorial design for medium optimization. All media are based on medium #65, which is SC medium prepared as described in the Material and Methods section. We have listed the concentration of each component for medium #65.

| **Medium** | Adenine | Alanine | Ammonium sulfate | Arginine | Asparagine | Aspartate | Biotin | Boric acid | Calcium Chloride | Calcium panthothenate | Copper sulfate | Cysteine | Ferric chloride | Folic acid | Glutamate | Glutamine | Glycine | Histidine | inositol |
| --- | --- | --- | --- | --- | --- | --- | --- | --- | --- | --- | --- | --- | --- | --- | --- | --- | --- | --- | --- |
| **1** | -1 | -1 | -1 | 1 | 1 | 1 | -1 | 1 | 1 | 1 | 1 | 1 | 1 | -1 | -1 | -1 | -1 | 1 | 1 |
| **2** | 1 | -1 | -1 | 1 | -1 | 1 | 1 | -1 | 1 | 1 | -1 | -1 | 1 | -1 | 1 | -1 | -1 | 1 | -1 |
| **3** | 1 | -1 | 1 | 1 | -1 | -1 | -1 | -1 | 1 | -1 | 1 | -1 | 1 | 1 | 1 | -1 | 1 | 1 | -1 |
| **4** | 1 | 1 | 1 | 1 | 1 | 1 | 1 | 1 | 1 | 1 | 1 | 1 | 1 | 1 | 1 | 1 | 1 | 1 | 1 |
| **5** | -1 | 1 | 1 | 1 | 1 | -1 | -1 | -1 | -1 | -1 | 1 | 1 | 1 | 1 | -1 | 1 | 1 | 1 | 1 |
| **6** | 1 | -1 | -1 | 1 | -1 | -1 | 1 | -1 | 1 | 1 | 1 | 1 | -1 | 1 | 1 | -1 | -1 | 1 | -1 |
| **7** | -1 | 1 | 1 | 1 | 1 | 1 | -1 | -1 | -1 | -1 | -1 | -1 | -1 | -1 | -1 | 1 | 1 | 1 | 1 |
| **8** | -1 | -1 | -1 | -1 | 1 | -1 | -1 | -1 | 1 | -1 | -1 | 1 | -1 | -1 | -1 | -1 | -1 | -1 | 1 |
| **9** | 1 | 1 | 1 | 1 | 1 | -1 | 1 | 1 | 1 | 1 | -1 | -1 | -1 | -1 | 1 | 1 | 1 | 1 | 1 |
| **10** | 1 | -1 | 1 | 1 | -1 | 1 | -1 | -1 | 1 | -1 | -1 | 1 | -1 | -1 | 1 | -1 | 1 | 1 | -1 |
| **11** | -1 | -1 | 1 | 1 | 1 | -1 | 1 | 1 | 1 | -1 | -1 | 1 | 1 | 1 | -1 | -1 | 1 | 1 | 1 |
| **12** | 1 | -1 | -1 | 1 | 1 | 1 | 1 | -1 | -1 | -1 | -1 | -1 | -1 | 1 | 1 | -1 | -1 | 1 | 1 |
| **13** | -1 | -1 | 1 | 1 | 1 | 1 | 1 | 1 | 1 | -1 | 1 | -1 | -1 | -1 | -1 | -1 | 1 | 1 | 1 |
| **14** | 1 | -1 | 1 | -1 | -1 | 1 | -1 | 1 | 1 | 1 | -1 | -1 | -1 | 1 | 1 | -1 | 1 | -1 | -1 |
| **15** | -1 | -1 | -1 | -1 | -1 | -1 | -1 | -1 | -1 | 1 | -1 | 1 | 1 | 1 | -1 | -1 | -1 | -1 | -1 |
| **16** | -1 | -1 | 1 | 1 | -1 | 1 | 1 | 1 | -1 | 1 | 1 | -1 | 1 | 1 | -1 | -1 | 1 | 1 | -1 |
| **17** | 1 | -1 | 1 | -1 | 1 | -1 | -1 | 1 | -1 | -1 | 1 | 1 | -1 | 1 | 1 | -1 | 1 | -1 | 1 |
| **18** | -1 | 1 | 1 | 1 | -1 | 1 | -1 | -1 | 1 | 1 | -1 | -1 | 1 | 1 | -1 | 1 | 1 | 1 | -1 |
| **19** | 1 | -1 | 1 | -1 | -1 | -1 | -1 | 1 | 1 | 1 | 1 | 1 | 1 | -1 | 1 | -1 | 1 | -1 | -1 |
| **20** | 1 | 1 | -1 | -1 | -1 | 1 | -1 | -1 | -1 | -1 | 1 | 1 | 1 | 1 | 1 | 1 | -1 | -1 | -1 |
| **21** | 1 | 1 | -1 | -1 | 1 | -1 | -1 | -1 | 1 | 1 | -1 | -1 | 1 | 1 | 1 | 1 | -1 | -1 | 1 |
| **22** | 1 | 1 | 1 | -1 | -1 | 1 | 1 | -1 | -1 | 1 | 1 | -1 | -1 | 1 | 1 | 1 | 1 | -1 | -1 |
| **23** | -1 | -1 | -1 | -1 | 1 | 1 | -1 | -1 | 1 | -1 | 1 | -1 | 1 | 1 | -1 | -1 | -1 | -1 | 1 |
| **24** | -1 | 1 | 1 | -1 | 1 | 1 | -1 | 1 | -1 | 1 | -1 | 1 | -1 | 1 | -1 | 1 | 1 | -1 | 1 |
| **25** | -1 | -1 | -1 | -1 | -1 | 1 | -1 | -1 | -1 | 1 | 1 | -1 | -1 | -1 | -1 | -1 | -1 | -1 | -1 |
| **26** | 1 | -1 | -1 | -1 | -1 | -1 | 1 | 1 | 1 | -1 | 1 | -1 | -1 | -1 | 1 | -1 | -1 | -1 | -1 |
| **27** | 1 | 1 | -1 | -1 | 1 | 1 | -1 | -1 | 1 | 1 | 1 | 1 | -1 | -1 | 1 | 1 | -1 | -1 | 1 |
| **28** | -1 | 1 | 1 | -1 | -1 | -1 | -1 | 1 | 1 | -1 | 1 | -1 | -1 | 1 | -1 | 1 | 1 | -1 | -1 |
| **29** | -1 | 1 | -1 | 1 | 1 | 1 | 1 | -1 | -1 | 1 | -1 | 1 | 1 | -1 | -1 | 1 | -1 | 1 | 1 |
| **30** | 1 | 1 | -1 | 1 | 1 | 1 | -1 | 1 | 1 | -1 | 1 | -1 | -1 | 1 | 1 | 1 | -1 | 1 | 1 |
| **31** | 1 | -1 | -1 | -1 | 1 | 1 | 1 | 1 | -1 | 1 | -1 | 1 | -1 | -1 | 1 | -1 | -1 | -1 | 1 |
| **32** | -1 | -1 | 1 | 1 | -1 | -1 | 1 | 1 | -1 | 1 | -1 | 1 | -1 | -1 | -1 | -1 | 1 | 1 | -1 |
| **33** | 1 | 1 | -1 | 1 | 1 | -1 | -1 | 1 | 1 | -1 | -1 | 1 | 1 | -1 | 1 | 1 | -1 | 1 | 1 |
| **34** | 1 | -1 | -1 | -1 | -1 | 1 | 1 | 1 | 1 | -1 | -1 | 1 | 1 | 1 | 1 | -1 | -1 | -1 | -1 |
| **35** | 1 | 1 | 1 | -1 | -1 | -1 | 1 | -1 | -1 | 1 | -1 | 1 | 1 | -1 | 1 | 1 | 1 | -1 | -1 |
| **36** | -1 | -1 | 1 | -1 | 1 | 1 | 1 | -1 | 1 | 1 | 1 | 1 | -1 | 1 | -1 | -1 | 1 | -1 | 1 |
| **37** | 1 | 1 | 1 | 1 | -1 | 1 | 1 | 1 | -1 | -1 | 1 | 1 | -1 | -1 | 1 | 1 | 1 | 1 | -1 |
| **38** | -1 | -1 | 1 | -1 | -1 | 1 | 1 | -1 | -1 | -1 | 1 | 1 | 1 | -1 | -1 | -1 | 1 | -1 | -1 |
| **39** | 1 | -1 | 1 | -1 | 1 | 1 | -1 | 1 | -1 | -1 | -1 | -1 | 1 | -1 | 1 | -1 | 1 | -1 | 1 |
| **40** | 1 | -1 | 1 | 1 | 1 | 1 | -1 | -1 | -1 | 1 | -1 | 1 | 1 | 1 | 1 | -1 | 1 | 1 | 1 |
| **41** | -1 | 1 | 1 | 1 | -1 | -1 | -1 | -1 | 1 | 1 | 1 | 1 | -1 | -1 | -1 | 1 | 1 | 1 | -1 |
| **42** | -1 | -1 | 1 | -1 | -1 | -1 | 1 | -1 | -1 | -1 | -1 | -1 | -1 | 1 | -1 | -1 | 1 | -1 | -1 |
| **43** | -1 | 1 | 1 | -1 | 1 | -1 | -1 | 1 | -1 | 1 | 1 | -1 | 1 | -1 | -1 | 1 | 1 | -1 | 1 |
| **44** | 1 | -1 | 1 | 1 | 1 | -1 | -1 | -1 | -1 | 1 | 1 | -1 | -1 | -1 | 1 | -1 | 1 | 1 | 1 |
| **45** | -1 | -1 | 1 | -1 | 1 | -1 | 1 | -1 | 1 | 1 | -1 | -1 | 1 | -1 | -1 | -1 | 1 | -1 | 1 |
| **46** | -1 | 1 | -1 | -1 | 1 | 1 | 1 | 1 | -1 | -1 | -1 | -1 | 1 | 1 | -1 | 1 | -1 | -1 | 1 |
| **47** | -1 | -1 | -1 | 1 | -1 | 1 | -1 | 1 | -1 | -1 | 1 | 1 | -1 | 1 | -1 | -1 | -1 | 1 | -1 |
| **48** | 1 | -1 | -1 | -1 | 1 | -1 | 1 | 1 | -1 | 1 | 1 | -1 | 1 | 1 | 1 | -1 | -1 | -1 | 1 |
| **49** | 1 | 1 | -1 | 1 | -1 | -1 | -1 | 1 | -1 | 1 | -1 | 1 | -1 | 1 | 1 | 1 | -1 | 1 | -1 |
| **50** | 1 | 1 | 1 | -1 | 1 | 1 | 1 | -1 | 1 | -1 | 1 | -1 | 1 | -1 | 1 | 1 | 1 | -1 | 1 |
| **51** | -1 | 1 | -1 | 1 | -1 | -1 | 1 | -1 | 1 | -1 | 1 | -1 | 1 | -1 | -1 | 1 | -1 | 1 | -1 |
| **52** | 1 | 1 | 1 | 1 | -1 | -1 | 1 | 1 | -1 | -1 | -1 | -1 | 1 | 1 | 1 | 1 | 1 | 1 | -1 |
| **53** | 1 | 1 | -1 | -1 | -1 | -1 | -1 | -1 | -1 | -1 | -1 | -1 | -1 | -1 | 1 | 1 | -1 | -1 | -1 |
| **54** | -1 | -1 | -1 | 1 | -1 | -1 | -1 | 1 | -1 | -1 | -1 | -1 | 1 | -1 | -1 | -1 | -1 | 1 | -1 |
| **55** | -1 | 1 | 1 | -1 | -1 | 1 | -1 | 1 | 1 | -1 | -1 | 1 | 1 | -1 | -1 | 1 | 1 | -1 | -1 |
| **56** | 1 | 1 | -1 | 1 | -1 | 1 | -1 | 1 | -1 | 1 | 1 | -1 | 1 | -1 | 1 | 1 | -1 | 1 | -1 |
| **57** | 1 | -1 | -1 | 1 | 1 | -1 | 1 | -1 | -1 | -1 | 1 | 1 | 1 | -1 | 1 | -1 | -1 | 1 | 1 |
| **58** | -1 | 1 | -1 | -1 | -1 | -1 | 1 | 1 | 1 | 1 | 1 | 1 | 1 | 1 | -1 | 1 | -1 | -1 | -1 |
| **59** | -1 | 1 | -1 | -1 | 1 | -1 | 1 | 1 | -1 | -1 | 1 | 1 | -1 | -1 | -1 | 1 | -1 | -1 | 1 |
| **60** | -1 | 1 | -1 | 1 | 1 | -1 | 1 | -1 | -1 | 1 | 1 | -1 | -1 | 1 | -1 | 1 | -1 | 1 | 1 |
| **61** | -1 | 1 | -1 | 1 | -1 | 1 | 1 | -1 | 1 | -1 | -1 | 1 | -1 | 1 | -1 | 1 | -1 | 1 | -1 |
| **62** | -1 | 1 | -1 | -1 | -1 | 1 | 1 | 1 | 1 | 1 | -1 | -1 | -1 | -1 | -1 | 1 | -1 | -1 | -1 |
| **63** | 1 | 1 | 1 | -1 | 1 | -1 | 1 | -1 | 1 | -1 | -1 | 1 | -1 | 1 | 1 | 1 | 1 | -1 | 1 |
| **64** | -1 | -1 | -1 | 1 | 1 | -1 | -1 | 1 | 1 | 1 | -1 | -1 | -1 | 1 | -1 | -1 | -1 | 1 | 1 |
| **65** | **18 mg/L** | **76 mg/L** | **5.0 g/L** | **76 mg/L** | **76 mg/L** | **76 mg/L** | **2.0 µg/L** | **500 µg/L** | **0.1 g/L** | **400 µg/L** | **40 µg/L** | **76 mg/L** | **200 µg/L** | **2.0 µg/L** | **76 mg/L** | **76 mg/L** | **76 mg/L** | **76 mg/L** | **2.76 mg/L** |

| **Medium** | Isoleucine | Leucine | Lysine | Magnesium Sulphate | Manganese sulfate | Methionine | Nicotinic acid | p-amino benzoic acid | Phenylalanine | Potassium Iodide | Potassium Phosphate | Proline | Pyridoxine HCl | Riboflavin, | Serine | Sodium Chloride | Sodium Molybdate | Thiamine HCL |
| --- | --- | --- | --- | --- | --- | --- | --- | --- | --- | --- | --- | --- | --- | --- | --- | --- | --- | --- |
| **1** | 1 | -1 | 1 | 1 | 1 | 1 | 1 | 1 | -1 | -1 | -1 | -1 | 1 | 1 | 1 | -1 | 1 | 1 |
| **2** | 1 | 1 | -1 | 1 | 1 | -1 | -1 | 1 | -1 | 1 | -1 | -1 | 1 | -1 | 1 | 1 | -1 | 1 |
| **3** | -1 | -1 | -1 | 1 | -1 | 1 | -1 | 1 | 1 | 1 | -1 | 1 | 1 | -1 | -1 | -1 | -1 | 1 |
| **4** | 1 | 1 | 1 | 1 | 1 | 1 | 1 | 1 | 1 | 1 | 1 | 1 | 1 | 1 | 1 | 1 | 1 | 1 |
| **5** | -1 | -1 | -1 | -1 | -1 | 1 | 1 | 1 | 1 | -1 | 1 | 1 | 1 | 1 | -1 | -1 | -1 | -1 |
| **6** | -1 | 1 | -1 | 1 | 1 | 1 | 1 | -1 | 1 | 1 | -1 | -1 | 1 | -1 | -1 | 1 | -1 | 1 |
| **7** | 1 | -1 | -1 | -1 | -1 | -1 | -1 | -1 | -1 | -1 | 1 | 1 | 1 | 1 | 1 | -1 | -1 | -1 |
| **8** | -1 | -1 | -1 | 1 | -1 | -1 | 1 | -1 | -1 | -1 | -1 | -1 | -1 | 1 | -1 | -1 | -1 | 1 |
| **9** | -1 | 1 | 1 | 1 | 1 | -1 | -1 | -1 | -1 | 1 | 1 | 1 | 1 | 1 | -1 | 1 | 1 | 1 |
| **10** | 1 | -1 | -1 | 1 | -1 | -1 | 1 | -1 | -1 | 1 | -1 | 1 | 1 | -1 | 1 | -1 | -1 | 1 |
| **11** | -1 | 1 | 1 | 1 | -1 | -1 | 1 | 1 | 1 | -1 | -1 | 1 | 1 | 1 | -1 | 1 | 1 | 1 |
| **12** | 1 | 1 | -1 | -1 | -1 | -1 | -1 | -1 | 1 | 1 | -1 | -1 | 1 | 1 | 1 | 1 | -1 | -1 |
| **13** | 1 | 1 | 1 | 1 | -1 | 1 | -1 | -1 | -1 | -1 | -1 | 1 | 1 | 1 | 1 | 1 | 1 | 1 |
| **14** | 1 | -1 | 1 | 1 | 1 | -1 | -1 | -1 | 1 | 1 | -1 | 1 | -1 | -1 | 1 | -1 | 1 | 1 |
| **15** | -1 | -1 | -1 | -1 | 1 | -1 | 1 | 1 | 1 | -1 | -1 | -1 | -1 | -1 | -1 | -1 | -1 | -1 |
| **16** | 1 | 1 | 1 | -1 | 1 | 1 | -1 | 1 | 1 | -1 | -1 | 1 | 1 | -1 | 1 | 1 | 1 | -1 |
| **17** | -1 | -1 | 1 | -1 | -1 | 1 | 1 | -1 | 1 | 1 | -1 | 1 | -1 | 1 | -1 | -1 | 1 | -1 |
| **18** | 1 | -1 | -1 | 1 | 1 | -1 | -1 | 1 | 1 | -1 | 1 | 1 | 1 | -1 | 1 | -1 | -1 | 1 |
| **19** | -1 | -1 | 1 | 1 | 1 | 1 | 1 | 1 | -1 | 1 | -1 | 1 | -1 | -1 | -1 | -1 | 1 | 1 |
| **20** | 1 | -1 | -1 | -1 | -1 | 1 | 1 | 1 | 1 | 1 | 1 | -1 | -1 | -1 | 1 | -1 | -1 | -1 |
| **21** | -1 | -1 | -1 | 1 | 1 | -1 | -1 | 1 | 1 | 1 | 1 | -1 | -1 | 1 | -1 | -1 | -1 | 1 |
| **22** | 1 | 1 | -1 | -1 | 1 | 1 | -1 | -1 | 1 | 1 | 1 | 1 | -1 | -1 | 1 | 1 | -1 | -1 |
| **23** | 1 | -1 | -1 | 1 | -1 | 1 | -1 | 1 | 1 | -1 | -1 | -1 | -1 | 1 | 1 | -1 | -1 | 1 |
| **24** | 1 | -1 | 1 | -1 | 1 | -1 | 1 | -1 | 1 | -1 | 1 | 1 | -1 | 1 | 1 | -1 | 1 | -1 |
| **25** | 1 | -1 | -1 | -1 | 1 | 1 | -1 | -1 | -1 | -1 | -1 | -1 | -1 | -1 | 1 | -1 | -1 | -1 |
| **26** | -1 | 1 | 1 | 1 | -1 | 1 | -1 | -1 | -1 | 1 | -1 | -1 | -1 | -1 | -1 | 1 | 1 | 1 |
| **27** | 1 | -1 | -1 | 1 | 1 | 1 | 1 | -1 | -1 | 1 | 1 | -1 | -1 | 1 | 1 | -1 | -1 | 1 |
| **28** | -1 | -1 | 1 | 1 | -1 | 1 | -1 | -1 | 1 | -1 | 1 | 1 | -1 | -1 | -1 | -1 | 1 | 1 |
| **29** | 1 | 1 | -1 | -1 | 1 | -1 | 1 | 1 | -1 | -1 | 1 | -1 | 1 | 1 | 1 | 1 | -1 | -1 |
| **30** | 1 | -1 | 1 | 1 | -1 | 1 | -1 | -1 | 1 | 1 | 1 | -1 | 1 | 1 | 1 | -1 | 1 | 1 |
| **31** | 1 | 1 | 1 | -1 | 1 | -1 | 1 | -1 | -1 | 1 | -1 | -1 | -1 | 1 | 1 | 1 | 1 | -1 |
| **32** | -1 | 1 | 1 | -1 | 1 | -1 | 1 | -1 | -1 | -1 | -1 | 1 | 1 | -1 | -1 | 1 | 1 | -1 |
| **33** | -1 | -1 | 1 | 1 | -1 | -1 | 1 | 1 | -1 | 1 | 1 | -1 | 1 | 1 | -1 | -1 | 1 | 1 |
| **34** | 1 | 1 | 1 | 1 | -1 | -1 | 1 | 1 | 1 | 1 | -1 | -1 | -1 | -1 | 1 | 1 | 1 | 1 |
| **35** | -1 | 1 | -1 | -1 | 1 | -1 | 1 | 1 | -1 | 1 | 1 | 1 | -1 | -1 | -1 | 1 | -1 | -1 |
| **36** | 1 | 1 | -1 | 1 | 1 | 1 | 1 | -1 | 1 | -1 | -1 | 1 | -1 | 1 | 1 | 1 | -1 | 1 |
| **37** | 1 | 1 | 1 | -1 | -1 | 1 | 1 | -1 | -1 | 1 | 1 | 1 | 1 | -1 | 1 | 1 | 1 | -1 |
| **38** | 1 | 1 | -1 | -1 | -1 | 1 | 1 | 1 | -1 | -1 | -1 | 1 | -1 | -1 | 1 | 1 | -1 | -1 |
| **39** | 1 | -1 | 1 | -1 | -1 | -1 | -1 | 1 | -1 | 1 | -1 | 1 | -1 | 1 | 1 | -1 | 1 | -1 |
| **40** | 1 | -1 | -1 | -1 | 1 | -1 | 1 | 1 | 1 | 1 | -1 | 1 | 1 | 1 | 1 | -1 | -1 | -1 |
| **41** | -1 | -1 | -1 | 1 | 1 | 1 | 1 | -1 | -1 | -1 | 1 | 1 | 1 | -1 | -1 | -1 | -1 | 1 |
| **42** | -1 | 1 | -1 | -1 | -1 | -1 | -1 | -1 | 1 | -1 | -1 | 1 | -1 | -1 | -1 | 1 | -1 | -1 |
| **43** | -1 | -1 | 1 | -1 | 1 | 1 | -1 | 1 | -1 | -1 | 1 | 1 | -1 | 1 | -1 | -1 | 1 | -1 |
| **44** | -1 | -1 | -1 | -1 | 1 | 1 | -1 | -1 | -1 | 1 | -1 | 1 | 1 | 1 | -1 | -1 | -1 | -1 |
| **45** | -1 | 1 | -1 | 1 | 1 | -1 | -1 | 1 | -1 | -1 | -1 | 1 | -1 | 1 | -1 | 1 | -1 | 1 |
| **46** | 1 | 1 | 1 | -1 | -1 | -1 | -1 | 1 | 1 | -1 | 1 | -1 | -1 | 1 | 1 | 1 | 1 | -1 |
| **47** | 1 | -1 | 1 | -1 | -1 | 1 | 1 | -1 | 1 | -1 | -1 | -1 | 1 | -1 | 1 | -1 | 1 | -1 |
| **48** | -1 | 1 | 1 | -1 | 1 | 1 | -1 | 1 | 1 | 1 | -1 | -1 | -1 | 1 | -1 | 1 | 1 | -1 |
| **49** | -1 | -1 | 1 | -1 | 1 | -1 | 1 | -1 | 1 | 1 | 1 | -1 | 1 | -1 | -1 | -1 | 1 | -1 |
| **50** | 1 | 1 | -1 | 1 | -1 | 1 | -1 | 1 | -1 | 1 | 1 | 1 | -1 | 1 | 1 | 1 | -1 | 1 |
| **51** | -1 | 1 | -1 | 1 | -1 | 1 | -1 | 1 | -1 | -1 | 1 | -1 | 1 | -1 | -1 | 1 | -1 | 1 |
| **52** | -1 | 1 | 1 | -1 | -1 | -1 | -1 | 1 | 1 | 1 | 1 | 1 | 1 | -1 | -1 | 1 | 1 | -1 |
| **53** | -1 | -1 | -1 | -1 | -1 | -1 | -1 | -1 | -1 | 1 | 1 | -1 | -1 | -1 | -1 | -1 | -1 | -1 |
| **54** | -1 | -1 | 1 | -1 | -1 | -1 | -1 | 1 | -1 | -1 | -1 | -1 | 1 | -1 | -1 | -1 | 1 | -1 |
| **55** | 1 | -1 | 1 | 1 | -1 | -1 | 1 | 1 | -1 | -1 | 1 | 1 | -1 | -1 | 1 | -1 | 1 | 1 |
| **56** | 1 | -1 | 1 | -1 | 1 | 1 | -1 | 1 | -1 | 1 | 1 | -1 | 1 | -1 | 1 | -1 | 1 | -1 |
| **57** | -1 | 1 | -1 | -1 | -1 | 1 | 1 | 1 | -1 | 1 | -1 | -1 | 1 | 1 | -1 | 1 | -1 | -1 |
| **58** | -1 | 1 | 1 | 1 | 1 | 1 | 1 | 1 | 1 | -1 | 1 | -1 | -1 | -1 | -1 | 1 | 1 | 1 |
| **59** | -1 | 1 | 1 | -1 | -1 | 1 | 1 | -1 | -1 | -1 | 1 | -1 | -1 | 1 | -1 | 1 | 1 | -1 |
| **60** | -1 | 1 | -1 | -1 | 1 | 1 | -1 | -1 | 1 | -1 | 1 | -1 | 1 | 1 | -1 | 1 | -1 | -1 |
| **61** | 1 | 1 | -1 | 1 | -1 | -1 | 1 | -1 | 1 | -1 | 1 | -1 | 1 | -1 | 1 | 1 | -1 | 1 |
| **62** | 1 | 1 | 1 | 1 | 1 | -1 | -1 | -1 | -1 | -1 | 1 | -1 | -1 | -1 | 1 | 1 | 1 | 1 |
| **63** | -1 | 1 | -1 | 1 | -1 | -1 | 1 | -1 | 1 | 1 | 1 | 1 | -1 | 1 | -1 | 1 | -1 | 1 |
| **64** | -1 | -1 | 1 | 1 | 1 | -1 | -1 | -1 | 1 | -1 | -1 | -1 | 1 | 1 | -1 | -1 | 1 | 1 |
| **65** | **76 mg/L** | **380 mg/L** | **76 mg/L** | **0.5 g/L** | **400 µg/L** | **76 mg/L** | **400 µg/L** | **8.2 mg/L** | **76 mg/L** | **100 µg/L** | **1.0 g/L** | **mg/L** | **400 µg/L** | **200 µg/L** | **76 mg/L** | **0.1 g/L** | **200 µg/L** | **400 µg/L** |

| **Medium** | Threonine | Tryptophan | Tyrosine | uracil | Valine | Zinc sulfate |
| --- | --- | --- | --- | --- | --- | --- |
| **1** | 1 | 1 | 1 | 1 | -1 | -1 |
| **2** | 1 | -1 | -1 | 1 | -1 | 1 |
| **3** | -1 | 1 | -1 | 1 | 1 | 1 |
| **4** | 1 | 1 | 1 | 1 | 1 | 1 |
| **5** | -1 | 1 | 1 | 1 | 1 | -1 |
| **6** | 1 | 1 | 1 | -1 | 1 | 1 |
| **7** | -1 | -1 | -1 | -1 | -1 | -1 |
| **8** | -1 | -1 | 1 | -1 | -1 | -1 |
| **9** | 1 | -1 | -1 | -1 | -1 | 1 |
| **10** | -1 | -1 | 1 | -1 | -1 | 1 |
| **11** | -1 | -1 | 1 | 1 | 1 | -1 |
| **12** | -1 | -1 | -1 | -1 | 1 | 1 |
| **13** | -1 | 1 | -1 | -1 | -1 | -1 |
| **14** | 1 | -1 | -1 | -1 | 1 | 1 |
| **15** | 1 | -1 | 1 | 1 | 1 | -1 |
| **16** | 1 | 1 | -1 | 1 | 1 | -1 |
| **17** | -1 | 1 | 1 | -1 | 1 | 1 |
| **18** | 1 | -1 | -1 | 1 | 1 | -1 |
| **19** | 1 | 1 | 1 | 1 | -1 | 1 |
| **20** | -1 | 1 | 1 | 1 | 1 | 1 |
| **21** | 1 | -1 | -1 | 1 | 1 | 1 |
| **22** | 1 | 1 | -1 | -1 | 1 | 1 |
| **23** | -1 | 1 | -1 | 1 | 1 | -1 |
| **24** | 1 | -1 | 1 | -1 | 1 | -1 |
| **25** | 1 | 1 | -1 | -1 | -1 | -1 |
| **26** | -1 | 1 | -1 | -1 | -1 | 1 |
| **27** | 1 | 1 | 1 | -1 | -1 | 1 |
| **28** | -1 | 1 | -1 | -1 | 1 | -1 |
| **29** | 1 | -1 | 1 | 1 | -1 | -1 |
| **30** | -1 | 1 | -1 | -1 | 1 | 1 |
| **31** | 1 | -1 | 1 | -1 | -1 | 1 |
| **32** | 1 | -1 | 1 | -1 | -1 | -1 |
| **33** | -1 | -1 | 1 | 1 | -1 | 1 |
| **34** | -1 | -1 | 1 | 1 | 1 | 1 |
| **35** | 1 | -1 | 1 | 1 | -1 | 1 |
| **36** | 1 | 1 | 1 | -1 | 1 | -1 |
| **37** | -1 | 1 | 1 | -1 | -1 | 1 |
| **38** | -1 | 1 | 1 | 1 | -1 | -1 |
| **39** | -1 | -1 | -1 | 1 | -1 | 1 |
| **40** | 1 | -1 | 1 | 1 | 1 | 1 |
| **41** | 1 | 1 | 1 | -1 | -1 | -1 |
| **42** | -1 | -1 | -1 | -1 | 1 | -1 |
| **43** | 1 | 1 | -1 | 1 | -1 | -1 |
| **44** | 1 | 1 | -1 | -1 | -1 | 1 |
| **45** | 1 | -1 | -1 | 1 | -1 | -1 |
| **46** | -1 | -1 | -1 | 1 | 1 | -1 |
| **47** | -1 | 1 | 1 | -1 | 1 | -1 |
| **48** | 1 | 1 | -1 | 1 | 1 | 1 |
| **49** | 1 | -1 | 1 | -1 | 1 | 1 |
| **50** | -1 | 1 | -1 | 1 | -1 | 1 |
| **51** | -1 | 1 | -1 | 1 | -1 | -1 |
| **52** | -1 | -1 | -1 | 1 | 1 | 1 |
| **53** | -1 | -1 | -1 | -1 | -1 | 1 |
| **54** | -1 | -1 | -1 | 1 | -1 | -1 |
| **55** | -1 | -1 | 1 | 1 | -1 | -1 |
| **56** | 1 | 1 | -1 | 1 | -1 | 1 |
| **57** | -1 | 1 | 1 | 1 | -1 | 1 |
| **58** | 1 | 1 | 1 | 1 | 1 | -1 |
| **59** | -1 | 1 | 1 | -1 | -1 | -1 |
| **60** | 1 | 1 | -1 | -1 | 1 | -1 |
| **61** | -1 | -1 | 1 | -1 | 1 | -1 |
| **62** | 1 | -1 | -1 | -1 | -1 | -1 |
| **63** | -1 | -1 | 1 | -1 | 1 | 1 |
| **64** | 1 | -1 | -1 | -1 | 1 | -1 |
| **65** | **76 mg/L** | **76 mg/L** | **76 mg/L** | **76 mg/L** | **76 mg/L** | **400 µg/L** |

**Supplementary table 9:** Identity score, query length and (query cover) for genes in organisms reported to produce ergothioneine when a BLASTp search was performed against the different Egt1, Egt2 and EgtE genes. Query cover percentage in brackets behind identity score. See supplementary table 10 for Genbank accession numbers.

|  | **NcasEgt1** | **CpurEgt1** | **SpomEgt1** | **RstoEgt1** | **AnidEgt1** | **AnigEgt1** | **ProqEgt1** |
| --- | --- | --- | --- | --- | --- | --- | --- |
| **NcasEgt1** |  | 61,  842 (95) | 34,  773 (92) | 36,  876 (92) | 50,  834 (97) | 50,  835 (97) | 51,  804 (95) |
| **CpurEgt1** |  |  | 33,  773 (92) | 36,  876 (92) | 51,  834 (96) | 49,  835 (97) | 49,  804 (95) |
| **SpomEgt1** |  |  |  | 33,  876 (97) | 32,  834 (96) | 32,  835 (97) | 33,  804 (97) |
|  |  |  |  |  |  |  |  |
|  | **PnotEgt1** | **SsalEgt1** | **PpolEgt1** | **AoryEgt1** | **AzinEgt1** | **AcarEgt1** | **MmucEgt1** |
| **NcasEgt1** | 49,  825 (97) | 31,  1032 (87) | - | 49,  845 (97) | - | 51,  837 (96) | - |
| **CpurEgt1** | 49,  825 (96) | 33,  1032 (87) | - | 48,  845 (96) | - | 49,  837 (97) | - |
| **SpomEgt1** | 32,  825 (97) | 30,  1032 (87) | - | 32,  845 (96) | - | 32,  837 (96) | - |
|  |  |  |  |  |  |  |  |
|  | **NtetEgt1** | **PpulEgt1** | **PostEgt1** | **PcitEgt1** | **LedoEgt1** | **GfroEgt1** | **GlucEgt1** |
| **NcasEgt1** | 98,  876 (100) | - | 34,  853 (87) | - | 34,  555 (57) | 37,  428 (57) | - |
| **CpurEgt1** | 61,  876 (96) | - | 35,  853 (95) | - | 34,  555 (55) | 39,  428 (57) | - |
| **SpomEgt1** | 33,  876 (97) | - | 33,  853 (96) | - | 36,  555 (53) | 38,  428 (55) | - |
|  |  |  |  |  |  |  |  |
|  | **HeriEgt1** | **AaegEgt1** | **CcibEgt1** | **MescEgt1** |  |  |  |
| **NcasEgt1** | - | - | - | - |  |  |  |
| **CpurEgt1** | - | - | - | - |  |  |  |
| **SpomEgt1** | - | - | - | - |  |  |  |
|  |  |  |  |  |  |  |  |
|  | **NcasEgt2** | **CpurEgt2** | **SpomEgt2** | **RstoEgt2** | **AnidEgt2** | **AnigEgt2** | **ProqEgt2** |
| **NcasEgt2** |  | 51,  526 (94) | 31,  392 (92) | 37,  408 (93) | 43,  470 (94) | 46,  450 (94) | 44,  585 (51) |
| **CpurEgt2** |  |  | 28,  392 (83) | 33,  408 (83) | 41,  470 (83) | 43,  450 (84) | 36,  585 (45) |
| **SpomEgt2** |  |  |  | 30,  408 (96) | 30,  470 (98) | 29,  450 (97) | 28,  585 (45) |
| **Table continued on next page** | | | | | | | |
|  | **PnotEgt2** | **SsalEgt2** | **PpolEgt2** | **AoryEgt2** | **AzinEgt2** | **AcarEgt2** | **MmucEgt2** |
| **NcasEgt2** | 43,  457 (94) | 37,  678 (92) | - | 45,  461 (93) | - | 43,  433 (90) | - |
| **CpurEgt2** | 37,  457 (84) | 39,  678 (82) | - | 42,  461 (83) | - | 40,  433 (79) | - |
| **SpomEgt2** | 28,  457 (99) | 28,  678 (96) | - | 27,  461 (97) | - | 29,  433 (92) | - |
|  |  |  |  |  |  |  |  |
|  | **NtetEgt2** | **PpulEgt2** | **PostEgt2** | **PcitEgt2** | **LedoEgt2** | **GfroEgt2** | **GlucEgt2** |
| **NcasEgt2** | 95,  473 (100) | - | 35,  445 (94) | - | 31,  581 (92) | 31,  439 (93) | 34,  427 (89) |
| **CpurEgt2** | 51,  473 (84) | - | 32,  445 (83) | - | 30,  581 (87) | 30,  439 (83) | 32,  427 (79) |
| **SpomEgt2** | 33,  473 (97) | - | 32,  445 (97) | - | 24,  581 (98) | 29,  439 (97) | 29,  427 (94) |
|  |  |  |  |  |  |  |  |
|  | **HeriEgt2** | **AaegEgt2** | **CcibEgt2** | **MescEgt2** |  |  |  |
| **NcasEgt2** | - | - | 41,  255 (5) | - |  |  |  |
| **CpurEgt2** | - | - | 37,  255 (3) | - |  |  |  |
| **SpomEgt2** | - | - | 46,  255 (3) | - |  |  |  |
|  |  |  |  |  |  |  |  |
|  | **MsmeEgtE** | **NastEgtE** | **SalbEgtE** | **SfraEgtE** | **SgriEgtE** | **AphiEgtE** | **AfumEgtE** |
| **MsmeEgtE** |  | 34,  396 (94) | 36,  512 (46) | 36,  371 (46) | 37,  463 (46) | 34,  381 (45) | 29,  517 (46) |
|  |  |  |  |  |  |  |  |
|  | **MturEgtE** | **MkanEgtE** | **MintEgtE** | **MforEgtE** | **MulcEgtE** | **MbalEgtE** | **MlepEgtE** |
| **MsmeEgtE** | 66,  390 (97) | 66,  378 (98) | 62,  385 (97) | 74,  371 (100) | 64,  383 (98) | 64,  383 (98) | 39,  82 (21) |
|  |  |  |  |  |  |  |  |
|  | **MaviEgtE** | **MbovEgtE** | **MmarEgtE** | **MmicEgtE** | **MparEgtE** | **MphlEgtE** | **MpisEgtE** |
| **MsmeEgtE** | 61,  381 (97) | 66,  390 (97) | 64,  383 (98) | 61,  375 (97) | 61,  381 (97) | 69,  371 (99) | - |
|  |  |  |  |  |  |  |  |
|  | **RrhoEgtE** | **AplaEgtE** | **AmaxEgtE** | **AfloEgtE** | ***Scytonema*** | ***Oscillatoria*** | ***Rhodophyta*** |
| **MsmeEgtE** | 34,  392 (99) | 28,  388 (45) | 31,  391 (38) | 29,  389 (64) | 28-40,  396  (21-59) | 28-30,  334-390  (46-59) | 29-36,  382-437  (46-52) |

**Supplementary table 10:** Fungal and bacterial organisms reported to produce ergothioneine in literature and their Egt1/Egt2/EgtE Genbank accession numbers found through homology searches.

| **Organism (fungi)** | **Reference** | **Egt1** | **Egt2** |
| --- | --- | --- | --- |
| *Neurospora crassa (Ncas)* | Genghof et al., 1956 | XP_956324.3 | XP_001728131.1 |
| *Claviceps purpurea (Cpur)* | Tanret, 1909 | CCE33591.1 | CCE33140.1 |
| *Schizosaccharomyces pombe (Spom)* | Pluskal et al., 2014 | NP_596639.2 | NP_595091.1 |
| *Rhizopus stolonifer (Rsto)* | Genghof, 1970 | RCH97401.1 | RCI05990.1 |
| *Aspergillus nidulans (Anid)* | Genghof, 1970 | XP_680889.1 | XP_663831.1 |
| *Aspergillus niger (Anig)* | Genghof, 1970 | XP_001397117.2 | XP_001390787.2 |
| *Penicillium roqueforti (Proq)* | Genghof, 1970 | CDM31097.1 | CDM34493.1 |
| *Penicillium notatum (Pnot)* | Genghof, 1970 | KZN88090.1 | KZN85331.1 |
| *Rhodotorula glutinis (Rglu)* | Genghof, 1970 | **Not found** | **Not found** |
| *Sporobolomyces salmonicolor (Ssal)* | Genghof, 1970 | CEQ42739.1 | CEQ41088.1 |
| *Physarum polycephalum (Ppol)* | Genghof, 1970 | **Not found** | **Not found** |
| *Aspergillus oryzae (Aory)* | Genghof et al., 1956 | XP_001727309.1 | XP_001821768.1 |
| *Alternaria zinnia (Azin)* | Genghof et al., 1956 | **Not found** | **Not found** |
| *Aspergillus carbonarius (Acar)* | Genghof et al., 1956 | OOF91620.1 | OOF99450.1 |
| *Mucor mucedo (Mmuc)* | Genghof et al., 1956 | **Not found** | **Not found** |
| *Neurospora tetrasperma (Ntet)* | Genghof et al., 1956 | XP_009849693.1 | XP_009848922.1 |
| *Pullularia pullulans (Ppul)* | Genghof et al., 1956 | **Not found** | **Not found** |
| *Agaricus bisporus (Abis)* | Kalaras et al., 2017 | XP_006462499.1 | XP_006461570.1 |
| *Pleurotus ostreatus (Post)* | Kalaras et al., 2017 | KDQ26018.1 | KDQ26326.1 |
| *Pleurotus citrinopileatus (Pcit)* | Kalaras et al., 2017 | **Not found** | **Not found** |
| *Lentinula edodes (Ledo)* | Kalaras et al., 2017 | GAW05586.1 | GAV99896.1 |
| *Grifola frondosa (Gfro)* | Kalaras et al., 2017 | OBZ71212.1 | OBZ72541.1 |
| *Ganoderma lucidum (Gluc)* | Kalaras et al., 2017 | **Not found** | AUN37957.1 |
| *Hericium erinaceus (Heri)* | Kalaras et al., 2017 | **Not found** | **Not found** |
| *Agrocybe aegerita (Aaeg)* | Kalaras et al., 2017 | **Not found** | **Not found** |
| *Cantharellus cibarius (Ccib)* | Kalaras et al., 2017 | **Not found** | AWA82152.1 |
| *Boletus edulis (Bedu)* | Kalaras et al., 2017 | **Not found** | **Not found** |
| *Morchella esculenta (Mesc)* | Kalaras et al., 2017 | **Not found** | **Not found** |
| **Organism (bacteria)** | **Reference** | **EgtE** |  |
| *Mycobacterium smegmatis (Msme)* | Seebeck, 2010 | ABK70212.1 |  |
| *Nocardia asteroids (Nast)* | Genghof, 1970 | SFL89244.1 |  |
| *Streptomyces albus (Salb)* | Genghof, 1970 | WP_030543061.1 |  |
| *Streptomyces fradiae (Sfra)* | Genghof, 1970 | WP_070159474.1 |  |
| *Streptomyces griseus (Sgri)* | Genghof, 1970 | WP_030852754.1 |  |
| *Actinoplanes philippinensis (Aphi)* | Genghof, 1970 | WP_093610803.1 |  |
| *Aspergillus fumigatus (Afum)* | Sheridan et al., 2016 | XP_754202.1 |  |
| *Mycobacterium tuberculosis (Mtur)* | Genghof and Vandamme, 1964 | WP_079029600.1 |  |
| *Mycobacterium kansasii (Mkan)* | Genghof and Vandamme, 1964 | WP_103802346.1 |  |
| *Mycobacterium intracellulare (Mint)* | Genghof and Vandamme, 1964 | WP_014941167.1 |  |
| *Mycobacterium forfuitum (Mfor)* | Genghof and Vandamme, 1964 | WP_076203140.1 |  |
| *Mycobacterium ulcerans (Mulc)* | Genghof and Vandamme, 1964 | WP_096369529.1 |  |
| *Mycobacterium balnei (Mbal)* | Genghof and Vandamme, 1964 | WP_117431391.1 |  |
| *Mycobacterium leprae (Mlep)* | Genghof and Vandamme, 1964 | WP_041323321.1 |  |
| *Mycobacterium avium (Mavi)* | Genghof and Vandamme, 1964 | WP_044543419.1 |  |
| *Mycobacterium bovis (Mbov)* | Genghof and Vandamme, 1964 | YP_009361087.1 |  |
| *Mycobacterium marinum (Mmar)* | Genghof and Vandamme, 1964 | WP_117431391.1 |  |
| *Mycobacterium microti (Mmic)* | Genghof and Vandamme, 1964 | PLV46245.1 |  |
| *Mycobacterium paratuberculosis (Mpar)* | Genghof and Vandamme, 1964 | AAS02619.1 |  |
| *Mycobacterium phlei (Mphl)* | Genghof and Vandamme, 1964 | WP_003888643.1 |  |
| *Mycobacterium piscinum (Mpis)* | Genghof and Vandamme, 1964 | **Not found** |  |
| *Rhodococcus rhodocrous (Rrho)*  ***Reclassified Mycobacterium rhodocrous*** | Genghof and Vandamme, 1964 | WP_006938916.1  *Multispecies* |  |
| *Arthrospira platensis (Apla)* | Pfeiffer et al., 2011 | WP_062945872.1 |  |
| *Arthrospira maxima (Amax)* | Pfeiffer et al., 2011 | WP_006621917.1  *Multispecies* |  |
| *Aphanizomenon flos-aquae (Aflo)* | Pfeiffer et al., 2011 | OBQ29810.1 |  |
| *Scytonema* sp.  **Genus wide search based on reference** | Pfeiffer et al., 2011 | WP_073633333.1  WP_096565387.1 |  |
| *Oscillatoria* sp.  **Genus wide search based on reference** | Pfeiffer et al., 2011 | WP_044196545.1  WP_015175683.1 |  |
| *Rhodophyta* sp.  **Genus wide search based on reference** | Pfeiffer et al., 2011 | OSX68822.1  PXF47457.1 |  |

**References**

Genghof, D. S. (1970). Biosynthesis of ergothioneine and hercynine by fungi and Actinomycetales. J. Bacteriol. 103, 475–8. Available at: http://www.ncbi.nlm.nih.gov/pubmed/5432011.

Genghof, D. S., Inamine, E., Kovalenko, V., and Melville, D. B. (1956). Ergothioneine in microorganisms. J. Biol. Chem. 223, 9–17. Available at: http://www.ncbi.nlm.nih.gov/pubmed/13376573.

Genghof, D. S., and Vandamme, O. (1964). Biosynthesis of ergothioneine and hercynine by mycobacteria. J. Bacteriol. 87, 852–62. Available at: http://www.ncbi.nlm.nih.gov/pubmed/14137624.

Jessop-Fabre, M. M., Jakočiūnas, T., Stovicek, V., Dai, Z., Jensen, M. K., Keasling, J. D., et al. (2016). EasyClone-MarkerFree: A vector toolkit for marker-less integration of genes into Saccharomyces cerevisiae via CRISPR-Cas9. Biotechnol. J. 11, 1110–1117. doi:10.1002/biot.201600147.

Kalaras, M. D., Richie, J. P., Calcagnotto, A., and Beelman, R. B. (2017). Mushrooms: A rich source of the antioxidants ergothioneine and glutathione. Food Chem. 233, 429–433. doi:10.1016/j.foodchem.2017.04.109.

Pfeiffer, C., Bauer, T., Surek, B., Schömig, E., and Gründemann, D. (2011). Cyanobacteria produce high levels of ergothioneine. Food Chem. 129, 1766–1769. doi:10.1016/j.foodchem.2011.06.047.

Pluskal, T., Ueno, M., and Yanagida, M. (2014). Genetic and metabolomic dissection of the ergothioneine and selenoneine biosynthetic pathway in the fission yeast, S. pombe, and construction of an overproduction system. PLoS One 9, e97774. doi:10.1371/journal.pone.0097774.

Seebeck, F. P. (2010). In vitro reconstitution of Mycobacterial ergothioneine biosynthesis. J. Am. Chem. Soc. 132, 6632–6633. doi:10.1021/ja101721e.

Sheridan, K. J., Lechner, B. E., Keeffe, G. O., Keller, M. A., Werner, E. R., Lindner, H., et al. (2016). Ergothioneine biosynthesis and functionality in the opportunistic fungal pathogen, Aspergillus fumigatus. Sci. Rep. 6, 35306. doi:10.1038/srep35306.

Skjoedt, M. L., Snoek, T., Kildegaard, K. R., Arsovska, D., Eichenberger, M., Goedecke, T. J., et al. (2016). Engineering prokaryotic transcriptional activators as metabolite biosensors in yeast. Nat. Chem. Biol. 12, 951–958. doi:10.1038/nchembio.2177.

Stovicek, V., Borodina, I., and Forster, J. (2015). CRISPR–Cas system enables fast and simple genome editing of industrial Saccharomyces cerevisiae strains. Metab. Eng. Commun. 2, 13–22. doi:10.1016/j.meteno.2015.03.001.

Tanret, C. (1909). The new base drawn from rye ergot, ergothioneine. C. R. Hebd. Acad. Sci., 222–224.
